# Supplementary material for: Face detection in untrained deep neural networks
Source: Nat Commun. 2021 Dec 16;12:7328. doi: 10.1038/s41467-021-27606-9 (PMC8677765; doi:10.1038/s41467-021-27606-9)
Supplement: Supplementary file 1 — Supplementary Information [file 41467_2021_27606_MOESM1_ESM.pdf]

Face Detection in Untrained Deep Neural Networks

Seungdae Baek[1 †], Min Song[2 †], Jaeson Jang[1], Gwangsu Kim[3], and Se-Bum Paik[1,2 *]

[1]Department of Bio and Brain Engineering, [2]Program of Brain and Cognitive Engineering, [3]Department of Physics, Korea Advanced Institute of Science and Technology, Daejeon 34141, Republic of Korea

† These authors contributed equally to this work

* Correspondence author: Se-Bum Paik (sbpaik@kaist.ac.kr)

Supplementary Materials

Layer	Type	Number of neurons	Kernels	Activations	RF size	RF size / total size (RF size / face size)
Input	Image input	227 × 227 × 3	Weights 11 × 11 × 3 × 96<br>Bias 1 × 1 × 96			
Conv1	Convolution	55 × 55 × 96		ReLU and cross channel normalization	11 × 11	2.35×10^{-3}<br>(7.39×10^{-3})
Pool1	Max pooling	27 × 27 × 96				
Conv2	Convolution	27 × 27× 256	Weights 5 × 5 × 48 × 256<br>Bias 1 × 1 × 256	ReLU and cross channel normalization	51 × 51	5.05×10^{-2}<br>(1.59×10^{-1})
Pool2	Max pooling	13 × 13 × 256				
Conv3	Convolution	13 × 13 × 384	Weights 3 × 3 × 256 × 384<br>Bias 1 × 1 × 384	ReLU	99 × 99	1.90×10^{-1}<br>(5.92×10^{-1})
Conv4	Convolution	13 × 13 × 384	Weights 3 × 3 × 192 × 384<br>Bias 1 × 1 × 384	ReLU	131 × 131	3.33×10^{-1}<br>(9.14×10^{-1})
Conv5	Convolution	13 × 13 × 256	Weights 3 × 3 × 192 × 256<br>Bias 1 × 1 × 256	ReLU	163 × 163	5.16×10^{-1}<br>(1.00)
Pool5	Max pooling	6 × 6 × 256				
FC6	Fully Connected	1 × 1 × 4096	Weights 4096 × 9216<br>Bias 4096 × 1	ReLU and dropout	227 × 227	1.00<br>(1.00)
FC7	Fully Connected	1 × 1 × 4096	Weights 4096 × 4096<br>Bias 4096 × 1	ReLU and dropout	227 × 227	1.00<br>(1.00)
FC8	Fully Connected	1 × 1 × 1000	Weights 1000 × 4096<br>Bias 1000 × 1	Softmax	227 × 227	1.00<br>(1.00)
Output	Classification Output					

Supplementary Table 1: Summary of the AlexNet Architecture

Panel	Group (n)		Test	*p* value	Effect size	Power
Figure 1						
f	FSI of face units (Conv2, n = 94)	FSI of shuffled response (Conv2, n = 94)	One-sided Rank-sum test	1.00	$r_{rbc} = -5.82 \times 10^{-1}$	2.04×10^{-2}
			One-sided Kolmogorov–Smirnov test	1.77×10^{-1}	$d = 2.49 \times 10^{-2}$	-
	FSI of face units (Conv3, n = 365)	FSI of shuffled response (Conv3, n = 365)	One-sided Rank-sum test	3.91×10^{-10}	$r_{rbc} = 2.89 \times 10^{-1}$	1.00
	FSI of face units (Conv4, n = 444)	FSI of shuffled response (Conv4, n = 444)	One-sided Rank-sum test	2.07×10^{-18}	$r_{rbc} = 3.47 \times 10^{-1}$	8.01×10^{-1}
	FSI of face units (Conv5, n = 465)	FSI of shuffled response (Conv5, n = 465)	One-sided Rank-sum test	1.49×10^{-25}	$r_{rbc} = 5.09 \times 10^{-1}$	1.00
	FSI of face units (Conv5, n = 465)	FSI of face neuron (Tsao 2010, n = 158)	Two-sided Rank-sum test	7.69×10^{-2}	$r_{rbc} = 9.25 \times 10^{-2}$	5.04×10^{-2}
			Two-sided Kolmogorov–Smirnov test	2.49×10^{-4}	$d = 2.32 \times 10^{-2}$	-
g	Response to face (n = 200)	Response to scrambled face (n = 200)	One-sided Rank-sum test	1.71×10^{-52}	$r_{rbc} = 7.69 \times 10^{-1}$	1.00
	Response to face (n = 200)	Response to texform face (n = 100)	One-sided Rank-sum test	4.12×10^{-30}	$r_{rbc} = 6.56 \times 10^{-1}$	1.00
	Response to non-face (n = 200)	Response to scrambled face (n = 100)	One-sided Rank-sum test	1.00	$r_{rbc} = -2.42 \times 10^{-1}$	1.11×10^{-19}
			One-sided Kolmogorov–Smirnov test	8.31×10^{-1}	$d = 3.00 \times 10^{-3}$	-
	Response to non-face (n = 200)	Response to texform face (n = 100)	One-sided Rank-sum test	9.40×10^{-1}	$r_{rbc} = -9.00 \times 10^{-2}$	3.48×10^{-7}
			One-sided Kolmogorov–Smirnov test	4.51×10^{-2}	$d = 1.84 \times 10^{-2}$	-
h	Response to face (n = 200)	Response to non-face (n = 200)	One-sided Rank-sum test	1.08×10^{-49}	$r_{rbc} = 7.40 \times 10^{-1}$	1.00
	Response to Tsao (2006, 2010) (n = 16)	Response to non-face (n = 200)	One-sided Rank-sum test	1.47×10^{-11}	$r_{rbc} = 4.54 \times 10^{-1}$	1.00
	Response to Cao (2018) (n = 50)	Response to non-face (n = 200)	One-sided Rank-sum test	8.95×10^{-15}	$r_{rbc} = 4.86 \times 10^{-1}$	1.00
	Response to Cao (2018, gray) (n = 50)	Response to non-face (n = 200)	One-sided Rank-sum test	1.91×10^{-19}	$r_{rbc} = 5.67 \times 10^{-1}$	1.00
	Response to FaceGen (n = 50)	Response to non-face (n = 200)	One-sided Rank-sum test	1.45×10^{-25}	$r_{rbc} = 6.58 \times 10^{-1}$	1.00
	Response to FaceGen (gray) (n = 50)	Response to non-face (n = 200)	One-sided Rank-sum test	3.32×10^{-26}	$r_{rbc} = 6.67 \times 10^{-1}$	1.00
Figure 2						
a	Response to Face PFI on last iteration (Reverse corr., n = 465)	Response to face stimulus (n = 465)	Two-sided Rank-sum test	1.51×10^{-6}	$r_{rbc} = 1.58 \times 10^{-1}$	8.62×10^{-1}
	Response to Face PFI on last iteration (Reverse corr., n = 465)	Response to non-face stimulus (n = 465)	Two-sided Rank-sum test	3.12×10^{-2}	$r_{rbc} = 7.07 \times 10^{-2}$	6.13×10^{-1}
b	Response to Face PFI on last iteration (X-Dream, n = 465)	Response to face stimulus (n = 465)	Two-sided Rank-sum test	1.92×10^{-119}	$r_{rbc} = 7.63 \times 10^{-1}$	1.00
	Response to Face PFI on last iteration (X-Dream, n = 465)	Response to non-face stimulus (n = 465)	Two-sided Rank-sum test	2.68×10^{-129}	$r_{rbc} = 7.94 \times 10^{-1}$	1.00
e	FCI of face stimulus (n = 200)	FCI of shuffled face PFI (Reverse corr., n = 465)	One-sided Rank-sum test	1.00	$r_{rbc} = 1.43 \times 10^{-1}$	5.39×10^{-2}

			One-sided Kolmogorov–Smirnov test	1.05 ×10^{-18}	d = 3.29 ×10^{-2}	-
	FCI of face PFI (Reverse corr., n = 465)	FCI of shuffled face PFI (Reverse corr., n = 465)	One-sided Rank-sum test	9.42 ×10^{-86}	r_{rbc} = 6.68 ×10^{-1}	1.00
	FCI of horn PFI (Reverse corr., n = 772)	FCI of shuffled face PFI (Reverse corr., n = 465)	One-sided Rank-sum test	1.88 ×10^{-1}	r_{rbc} = 3.18 ×10^{-2}	1.23 ×10^{-1}
			One-sided Kolmogorov–Smirnov test	2.70 ×10^{-42}	d = 2.67 ×10^{-2}	-
	FCI of hand PFI (Reverse corr., n = 7)	FCI of shuffled face PFI (Reverse corr., n = 465)	One-sided Rank-sum test	7.43 ×10^{-1}	r_{rbc} = -3.12 ×10^{-2}	2.34 ×10^{-2}
			One-sided Kolmogorov–Smirnov test	5.95 ×10^{-2}	d = 1.61 ×10^{-1}	-
	FCI of chair PFI (Reverse corr., n = 63)	FCI of shuffled face PFI (Reverse corr., n = 465)	One-sided Rank-sum test	1.00	r_{rbc} = -3.36 ×10^{-1}	2.95 ×10^{-16}
			One-sided Kolmogorov–Smirnov test	5.66 ×10^{-2}	d = 2.00 ×10^{-2}	-
	FCI of flower PFI (Reverse corr., n = 107)	FCI of shuffled face PFI (Reverse corr., n = 465)	One-sided Rank-sum test	1.00	r_{rbc} = -5.69 ×10^{-1}	6.73 ×10^{-60}
			One-sided Kolmogorov–Smirnov test	3.37 ×10^{-1}	d = 7.33 ×10^{-3}	-
	FCI of horn PFI (Reverse corr., n = 772)	FCI of face PFI (Reverse corr., n = 465)	One-sided Rank-sum test	1.46 ×10^{-39}	r_{rbc} = 4.71 ×10^{-1}	9.67 ×10^{-1}
	FCI of hand PFI (Reverse corr., n = 7)	FCI of face PFI (Reverse corr., n = 465)	One-sided Rank-sum test	5.63 ×10^{-4}	r_{rbc} = 1.56 ×10^{-1}	1.00
	FCI of chair PFI (Reverse corr., n = 63)	FCI of face PFI (Reverse corr., n = 465)	One-sided Rank-sum test	2.83 ×10^{-28}	r_{rbc} = 4.94 ×10^{-1}	1.00
	FCI of flower PFI (Reverse corr., n = 107)	FCI of face PFI (Reverse corr., n = 465)	One-sided Rank-sum test	1.19 ×10^{-51}	r_{rbc} = 6.51 ×10^{-1}	1.00
f	FCI of face PFI (X-Dream, n = 465)	FCI of shuffled face PFI (X-Dream, n = 465)	One-sided Rank-sum test	3.94 ×10^{-121}	r_{rbc} = 8.46 ×10^{-1}	1.00
	FCI of chair PFI (X-Dream, n = 63)	FCI of shuffled face PFI (X-Dream, n = 465)	One-sided Rank-sum test	9.96 ×10^{-1}	r_{rbc} = -1.12 ×10^{-1}	4.71 ×10^{-4}
			One-sided Kolmogorov–Smirnov test	4.34 ×10^{-8}	d = 4.84 ×10^{-2}	-
	FCI of horn PFI (X-Dream, n = 772)	FCI of shuffled face PFI (X-Dream, n = 465)	One-sided Rank-sum test	1.00	r_{rbc} = -3.14 ×10^{-1}	2.45 ×10^{-40}
			One-sided Kolmogorov–Smirnov test	1.24 ×10^{-24}	d = 1.10 ×10^{-2}	-
	FCI of hand PFI (X-Dream, n = 7)	FCI of shuffled face PFI (X-Dream, n = 465)	One-sided Rank-sum test	9.74 ×10^{-1}	r_{rbc} = -8.97 ×10^{-2}	1.86 ×10^{-4}
			One-sided Kolmogorov–Smirnov test	2.81 ×10^{-1}	d = 1.08 ×10^{-1}	-
	FCI of flower PFI (X-Dream, n = 107)	FCI of shuffled face PFI (X-Dream, n = 465)	One-sided Rank-sum test	1.00	r_{rbc} = -3.18 ×10^{-1}	1.18 ×10^{-15}
			One-sided Kolmogorov–Smirnov test	5.00 ×10^{-4}	d = 2.54 ×10^{-2}	-
	FCI of chair PFI (X-Dream, n = 63)	FCI of face PFI (X-Dream, n = 465)	One-sided Rank-sum test	3.59 ×10^{-23}	r_{rbc} = 5.18 ×10^{-1}	1.00
	FCI of horn PFI (X-Dream, n = 772)	FCI of face PFI (X-Dream, n = 465)	One-sided Rank-sum test	3.90 ×10^{-100}	r_{rbc} = 6.48 ×10^{-1}	1.00
	FCI of hand PFI (X-Dream, n = 7)	FCI of face PFI (X-Dream, n = 465)	One-sided Rank-sum test	1.93 ×10^{-5}	r_{rbc} = 2.35 ×10^{-1}	8.99 ×10^{-1}

	FCI of flower PFI (X-Dream, n = 107)	FCI of face PFI (X-Dream, n = 465)	One-sided Rank-sum test	3.75×10^{-38}	$r_{rbc} = 6.38 \times 10^{-1}$	1.00
Figure 3						
b	Performance using singe face unit (n = 465)	Performance using shuffled response (n = 465)	Two-sided Rank-sum test	2.97×10^{-121}	$r_{rbc} = 7.68 \times 10^{-1}$	1.00
	Performance using singe non-selective unit (n = 7,776)	Performance using shuffled response (n = 465)	Two-sided Rank-sum test	1.10×10^{-1}	$r_{rbc} = 4.52 \times 10^{-2}$	2.27×10^{-1}
			Two-sided Kolmogorov–Smirnov test	1.93×10^{-1}	$d = 2.12 \times 10^{-2}$	-
c	Performance using face units (n = 100)	Performance using non-selective units (n = 100)	Two-sided Rank-sum test	$\leq 1.45 \times 10^{-33}$	$r_{rbc} \geq 8.74 \times 10^{-1}$	1.00
d	Performance using face units (n = 100)	Performance using all units (n = 100)	Two-sided Rank-sum test	1.90×10^{-1}	$r_{rbc} = 9.29 \times 10^{-2}$	5.24×10^{-1}
			Kolmogorov–Smirnov test	1.90×10^{-1}	$d = 9.20 \times 10^{-3}$	-
Figure 4						
b	FSI of face unit on untrained net (n = 4,267)	FSI of face unit on face-reduced ImageNet net (n = 2,452)	Two-sided Rank-sum test	2.99×10^{-28}	$r_{rbc} = 6.76 \times 10^{-1}$	1.00
	FSI of face unit on untrained net (n = 4,267)	FSI of face unit on ImageNet trained net (n = 3,561)	Two-sided Rank-sum test	9.34×10^{-45}	$r_{rbc} = 8.15 \times 10^{-1}$	1.00
	FSI of face unit on untrained net (n = 4,267)	FSI of face unit on ImageNet +Face trained net (n = 3,585)	Two-sided Rank-sum test	1.21×10^{-3}	$r_{rbc} = 2.60 \times 10^{-1}$	9.62×10^{-1}
c	Number of face unit on untrained net (n = 10)	Number of face unit on face-reduced ImageNet net (n = 10)	Two-sided Rank-sum test	1.83×10^{-4}	$r_{rbc} = 8.67 \times 10^{-1}$	1.00
	Number of face unit on untrained net (n = 10)	Number of face unit on ImageNet trained net (n = 10)	Two-sided Rank-sum test	1.30×10^{-3}	$r_{rbc} = 7.46 \times 10^{-1}$	1.00
	Number of face unit on untrained net (n = 10)	Number of face unit on ImageNet +Face trained net (n = 10)	Two-sided Rank-sum test	1.40×10^{-3}	$r_{rbc} = 5.72 \times 10^{-1}$	9.99×10^{-1}
d	Performance of face unit on untrained net (n = 1,000)	Performance of face unit on face-reduced ImageNet net (n = 1,000)	Two-sided Rank-sum test	1.06×10^{-22}	$r_{rbc} = 6.42 \times 10^{-1}$	1.00
	Performance of face unit on untrained net (n = 1,000)	Performance of face unit on ImageNet trained net (n = 1,000)	Two-sided Rank-sum test	1.06×10^{-5}	$r_{rbc} = 3.30 \times 10^{-1}$	1.00
	Performance of face unit on untrained net (n = 1,000)	Performance of face unit on ImageNet+Face trained net (n = 1,000)	Two-sided Rank-sum test	1.04×10^{-3}	$r_{rbc} = 2.60 \times 10^{-1}$	8.32×10^{-1}
Figure 5						
b	Response to gazania (n = 100)	Response to scrambled gazania (n = 100)	One-sided Rank-sum test	2.67×10^{-12}	$r_{rbc} = 4.89 \times 10^{-1}$	1.00
	Response to gazania (n = 100)	Response to texform gazania (n = 100)	One-sided Rank-sum test	2.08×10^{-14}	$r_{rbc} = 5.36 \times 10^{-1}$	1.00
	Response to other class (n = 100)	Response to scrambled gazania (n = 100)	One-sided Rank-sum test	1.00	$r_{rbc} = 3.17 \times 10^{-1}$	1.00
			One-sided Kolmogorov–Smirnov test	1.02×10^{-2}	$d = 2.97 \times 10^{-2}$	-
	Response to other class (n = 100)	Response to texform gazania (n = 100)	One-sided Rank-sum test	1.00	$r_{rbc} = 4.20 \times 10^{-1}$	1.00
			One-sided Kolmogorov–Smirnov test	3.45×10^{-2}	$d = 2.55 \times 10^{-2}$	-

Supplementary Table 2: Summary of statistical tests on the main figure

Panel	Group (n)		Test	*p* value	Effect size	Power
Figure S1						
b	Luminance of face stimulus (n = 200)	Luminance of hand stimulus (n = 200)	Two-sided Rank-sum test	1.00	-	-
			Two-sided Kolmogorov–Smirnov test	1.00	-	-
	Luminance of face stimulus (n = 200)	Luminance of horn stimulus (n = 200)	Two-sided Rank-sum test	1.00	-	-
			Two-sided Kolmogorov–Smirnov test	1.00	-	-
	Luminance of face stimulus (n = 200)	Luminance of flower stimulus (n = 200)	Two-sided Rank-sum test	1.00	-	-
			Two-sided Kolmogorov–Smirnov test	1.00	-	-
	Luminance of face stimulus (n = 200)	Luminance of chair stimulus (n = 200)	Two-sided Rank-sum test	1.00	-	-
			Two-sided Kolmogorov–Smirnov test	1.00	-	-
	Luminance of face stimulus (n = 200)	Luminance of scrambled face stimulus (n = 200)	Two-sided Rank-sum test	1.00	-	-
			Two-sided Kolmogorov–Smirnov test	1.00	-	-
c	Contrast of face stimulus (n = 200)	Contrast of hand stimulus (n = 200)	Two-sided Rank-sum test	1.00	-	-
			Kolmogorov–Smirnov test	1.00	-	-
	Contrast of face stimulus (n = 200)	Contrast of horn stimulus (n = 200)	Two-sided Rank-sum test	1.00	-	-
			Two-sided Kolmogorov–Smirnov test	1.00	-	-
	Contrast of face stimulus (n = 200)	Contrast of flower stimulus (n = 200)	Two-sided Rank-sum test	1.00	-	-
			Two-sided Kolmogorov–Smirnov test	1.00	-	-
	Contrast of face stimulus (n = 200)	Contrast of chair stimulus (n = 200)	Two-sided Rank-sum test	1.00	-	-
			Two-sided Kolmogorov–Smirnov test	1.00	-	-
	Contrast of face stimulus (n = 200)	Contrast of scrambled face stimulus (n = 200)	Two-sided Rank-sum test	1.00	-	-
			Two-sided Kolmogorov–Smirnov test	1.00	-	-
d	Object size of face stimulus (n = 200)	Object size of hand stimulus (n = 200)	Two-sided Rank-sum test	9.43×10^{-1}	$r_{rbc} = 3.60 \times 10^{-3}$	5.61×10^{-2}
			Two-sided Kolmogorov–Smirnov test	8.33×10^{-1}	$d = 4.12 \times 10^{-3}$	-
	Object size of face stimulus (n = 200)	Object size of horn stimulus (n = 200)	Two-sided Rank-sum test	6.13×10^{-1}	$r_{rbc} = 2.54 \times 10^{-2}$	8.98×10^{-2}
			Two-sided Kolmogorov–Smirnov test	6.81×10^{-1}	$d = 3.32 \times 10^{-2}$	-

	Object size of face stimulus (n = 200)	Object size of flower stimulus (n = 200)	Two-sided Rank-sum test	3.22×10^{-1}	$r_{rbc} = 4.96 \times 10^{-2}$	2.40×10^{-1}
			Two-sided Kolmogorov–Smirnov test	2.98×10^{-1}	$d = 4.53 \times 10^{-2}$	-
	Object size of face stimulus (n = 200)	Object size of chair stimulus (n = 200)	Two-sided Rank-sum test	6.43×10^{-1}	$r_{rbc} = 2.33 \times 10^{-2}$	9.14×10^{-2}
			Two-sided Kolmogorov–Smirnov test	5.94×10^{-1}	$d = 2.12 \times 10^{-2}$	-
	Object size of face stimulus (n = 200)	Object size of scrambled face stimulus (n = 200)	Two-sided Rank-sum test	1.00	-	-
			Two-sided Kolmogorov–Smirnov test	1.00	-	-
e	Object location (x-axis) of face stimulus (n = 200)	Object location (x-axis) of hand stimulus (n = 200)	Two-sided Rank-sum test	5.16×10^{-1}	$r_{rbc} = -3.26 \times 10^{-2}$	1.53×10^{-1}
			Two-sided Kolmogorov–Smirnov test	4.78×10^{-1}	$d = 3.74 \times 10^{-2}$	-
	Object location (x-axis) of face stimulus (n = 200)	Object location (x-axis) of horn stimulus (n = 200)	Two-sided Rank-sum test	9.57×10^{-1}	$r_{rbc} = -2.74 \times 10^{-3}$	5.02×10^{-2}
			Two-sided Kolmogorov–Smirnov test	8.87×10^{-1}	$d = 5.10 \times 10^{-3}$	-
	Object location (x-axis) of face stimulus n = 200)	Object location (x-axis) of flower stimulus (n = 200)	Two-sided Rank-sum test	6.61×10^{-1}	$r_{rbc} = -2.20 \times 10^{-2}$	6.31×10^{-2}
			Two-sided Kolmogorov–Smirnov test	6.74×10^{-1}	$d = 3.11 \times 10^{-2}$	-
	Object location (x-axis) of face stimulus (n = 200)	Object location (x-axis) of chair stimulus (n = 200)	Two-sided Rank-sum test	8.99×10^{-1}	$r_{rbc} = -6.42 \times 10^{-3}$	6.25×10^{-2}
			Two-sided Kolmogorov–Smirnov test	9.01×10^{-1}	$d = 8.31 \times 10^{-3}$	-
	Object location (x-axis) of face stimulus (n = 200)	Object location (x-axis) of scrambled face stimulus (n = 200)	Two-sided Rank-sum test	1.00	-	-
			Two-sided Kolmogorov–Smirnov test	1.00	-	-
f	Intra-class image similarity of face stimulus (n = 19,900)	Object location (y-axis) of hand stimulus (n = 200)	Two-sided Rank-sum test	4.31×10^{-1}	$r_{rbc} = 3.94 \times 10^{-2}$	3.94×10^{-1}
			Two-sided Kolmogorov–Smirnov test	5.12×10^{-1}	$d = 4.31 \times 10^{-2}$	-
	Intra-class image similarity of face stimulus (n = 19,900)	Object location (y-axis) of horn stimulus (n = 200)	Two-sided Rank-sum test	4.29×10^{-1}	$r_{rbc} = 3.96 \times 10^{-2}$	2.01×10^{-1}
			Two-sided Kolmogorov–Smirnov test	4.83×10^{-1}	$d = 4.57 \times 10^{-2}$	-
	Object location (y-axis) of face stimulus n = 200)	Object location (y-axis) of flower stimulus (n = 200)	Two-sided Rank-sum test	8.99×10^{-1}	$r_{rbc} = 6.41 \times 10^{-3}$	7.58×10^{-2}
			Two-sided Kolmogorov–Smirnov test	8.35×10^{-1}	$d = 7.21 \times 10^{-3}$	-
	Object location (y-axis) of face stimulus (n = 200)	Object location (y-axis) of chair stimulus (n = 200)	Two-sided Rank-sum test	9.49×10^{-1}	$r_{rbc} = 3.23 \times 10^{-3}$	5.95×10^{-2}
			Two-sided Kolmogorov–Smirnov test	9.32×10^{-1}	$d = 4.72 \times 10^{-3}$	-
	Object location (y-axis) of face stimulus (n = 200)	Object location (x-axis) of scrambled face stimulus (n = 200)	Two-sided Rank-sum test	1.00	-	-
			Two-sided Kolmogorov–Smirnov test	1.00	-	-

g	Intra-class image similarity of face stimulus (n = 19,900)	Zero value (n = 1)	One-sided Signed-rank test	5.02×10^{-1}	$r_{rbc} = 2.84 \times 10^{-5}$	5.00×10^{-2}
			One-sided Kolmogorov–Smirnov test	4.11×10^{-1}	$d = 3.21 \times 10^{-2}$	-
	Intra-class image similarity of hand stimulus (n = 19,900)	Zero value (n = 1)	One-sided Signed-rank test	4.96×10^{-1}	$r_{rbc} = -6.54 \times 10^{-5}$	4.99×10^{-2}
			One-sided Kolmogorov–Smirnov test	4.43×10^{-1}	$d = 5.13 \times 10^{-2}$	-
	Intra-class image similarity of horn stimulus (n = 19,900)	Zero value (n = 1)	One-sided Signed-rank test	4.97×10^{-1}	$r_{rbc} = -6.29 \times 10^{-5}$	5.00×10^{-2}
			One-sided Kolmogorov–Smirnov test	5.13×10^{-1}	$d = 5.37 \times 10^{-2}$	-
	Intra-class image similarity of flower stimulus (n = 19,900)	Zero value (n = 1)	One-sided Signed-rank test	4.96×10^{-1}	$r_{rbc} = -7.16 \times 10^{-5}$	4.97×10^{-2}
			One-sided Kolmogorov–Smirnov test	5.68×10^{-1}	$d = 6.13 \times 10^{-2}$	-
	Intra-class image similarity of chair stimulus (n = 19,900)	Zero value (n = 1)	One-sided Signed-rank test	5.03×10^{-1}	$r_{rbc} = 5.55 \times 10^{-5}$	5.00×10^{-2}
			One-sided Kolmogorov–Smirnov test	6.75×10^{-1}	$d = 5.75 \times 10^{-2}$	-
	Intra-class image similarity of scrambled face stimulus (n =19,900)	Zero value (n = 1)	One-sided Signed-rank test	5.10×10^{-1}	$r_{rbc} = -1.73 \times 10^{-4}$	4.98×10^{-2}
			One-sided Kolmogorov–Smirnov test	4.84×10^{-1}	$d = 2.71 \times 10^{-2}$	-
	Intra-class image similarity of face stimulus (n = 19,900)	Intra-class image similarity of hand stimulus (n = 19,900)	Two-sided Rank-sum test	9.21×10^{-1}	$r_{rbc} = -4.95 \times 10^{-4}$	6.32×10^{-2}
			Two-sided Kolmogorov–Smirnov test	8.74×10^{-1}	$d = 5.19 \times 10^{-2}$	-
	Intra-class image similarity of face stimulus (n = 19900)	Intra-class image similarity of horn stimulus (n = 19,900)	Two-sided Rank-sum test	7.91×10^{-1}	$r_{rbc} = -1.29 \times 10^{-3}$	7.66×10^{-2}
			Two-sided Kolmogorov–Smirnov test	8.01×10^{-1}	$d = 1.68 \times 10^{-3}$	-
	Intra-class image similarity of face stimulus (n = 19,900)	Intra-class image similarity of flower stimulus (n = 19,900)	Two-sided Rank-sum test	9.39×10^{-1}	$r_{rbc} = -3.81 \times 10^{-4}$	6.79×10^{-2}
			Two-sided Kolmogorov–Smirnov test	8.73×10^{-1}	$d = 2.15 \times 10^{-2}$	-
	Intra-class image similarity of face stimulus (n = 19,900)	Intra-class image similarity of chair stimulus (n = 19,900)	Two-sided Rank-sum test	2.98×10^{-1}	$r_{rbc} = -5.19 \times 10^{-2}$	2.78×10^{-1}
			Two-sided Kolmogorov–Smirnov test	3.04×10^{-1}	$d = 4.74 \times 10^{-3}$	-
	Intra-class image similarity of face stimulus (n = 19,900)	Intra-class image similarity of scrambled face stimulus (n =19,900)	Two-sided Rank-sum test	4.38×10^{-1}	$r_{rbc} = -3.90 \times 10^{-3}$	1.70×10^{-1}
			Two-sided Kolmogorov–Smirnov test	4.12×10^{-1}	$d = 4.53 \times 10^{-2}$	-
h	Intra-class image similarity of face stimulus (n = 19,900)	Inter-class image similarity of hand stimulus (n = 20,100)	Two-sided Rank-sum test	5.50×10^{-1}	$r_{rbc} = 3.01 \times 10^{-3}$	8.08×10^{-2}
			Two-sided Kolmogorov–Smirnov test	5.67×10^{-1}	$d = 3.51 \times 10^{-2}$	-
	Intra-class image similarity of face stimulus (n = 19,900)	Inter-class image similarity of horn stimulus (n = 20,100)	Two-sided Rank-sum test	8.31×10^{-1}	$r_{rbc} = 1.07 \times 10^{-3}$	7.01×10^{-2}
			Two-sided Kolmogorov–Smirnov test	8.43×10^{-1}	$d = 2.74 \times 10^{-2}$	-

	Intra-class image similarity of face stimulus (n = 19,900)	Inter-class image similarity of flower stimulus (n = 20,100)	Two-sided Rank-sum test	5.71×10^{-1}	$r_{rbc} = 2.81 \times 10^{-3}$	9.97×10^{-2}
			Two-sided Kolmogorov–Smirnov test	5.97×10^{-1}	$d = 3.57 \times 10^{-2}$	-
	Intra-class image similarity of face stimulus (n = 19,900)	Intra-class image similarity of chair stimulus (n = 20,100)	Two-sided Rank-sum test	5.92×10^{-1}	$r_{rbc} = 2.66 \times 10^{-3}$	5.80×10^{-2}
			Two-sided Kolmogorov–Smirnov test	6.05×10^{-1}	$d = 3.15 \times 10^{-2}$	-
	Intra-class image similarity of face stimulus (n = 19,900)	Intra-class image similarity of scrambled face stimulus (n =20,100)	Two-sided Rank-sum test	7.41×10^{-1}	$r_{rbc} = 1.68 \times 10^{-3}$	5.00×10^{-2}
			Two-sided Kolmogorov–Smirnov test	7.13×10^{-1}	$d = 2.41 \times 10^{-2}$	-
Figure S2						
h	Number of face units in 1st group of Conv4 (n = 100)	Number of face units in 2nd group of Conv4 (n = 100)	Two-sided Rank-sum test	4.60×10^{-1}	$r_{rbc} = 5.25 \times 10^{-2}$	1.59×10^{-1}
			Two-sided Kolmogorov–Smirnov test	2.61×10^{-1}	$d = 7.83 \times 10^{-2}$	-
	Number of face units in 1st group of Conv5 (n = 100)	Number of face units in 2nd group of Conv5 (n = 100)	Two-sided Rank-sum test	5.44×10^{-1}	$r_{rbc} = 4.31 \times 10^{-2}$	5.05×10^{-2}
			Two-sided Kolmogorov–Smirnov test	5.56×10^{-1}	$d = 3.02 \times 10^{-2}$	-
Figure S3						
	FSI of face units (Aparicio, 2016; Conv5, n = 465)	FSI of face neuron (Aparicio, 2016; Tsao 2010, n = 158)	Two-sided Rank-sum test	7.69×10^{-2}	$r_{rbc} = 9.25 \times 10^{-2}$	5.04×10^{-2}
			Two-sided Kolmogorov–Smirnov test	2.49×10^{-4}	$d = 2.32 \times 10^{-2}$	-
	FSI of face units (Aparicio, 2016; Conv5, n = 465)	FSI of shuffled response (Aparicio, 2016; Conv5, n = 465)	One-sided Rank-sum test	1.49×10^{-25}	$r_{rbc} = 5.09 \times 10^{-1}$	1.00
	FSI of face units (Tsao 2006; Conv5, n = 465)	FSI of face neuron (Tsao 2006; Tsao 2010, n = 158)	Two-sided Rank-sum test	4.86×10^{-1}	$r_{rbc} = 3.84 \times 10^{-2}$	3.36×10^{-1}
			Two-sided Kolmogorov–Smirnov test	5.28×10^{-1}	$d = 9.67 \times 10^{-3}$	-
	FSI of face units (Tsao 2006; Conv5, n = 465)	FSI of shuffled response (Tsao 2006; Conv5, n = 465)	One-sided Rank-sum test	4.65×10^{-5}	$r_{rbc} = 2.10 \times 10^{-1}$	6.68×10^{-1}
	FSI of face units (Duyck 2021; Conv5, n = 465)	FSI of face neuron (Duyck 2021; Tsao 2010, n = 158)	Two-sided Rank-sum test	9.48×10^{-1}	$r_{rbc} = 3.44 \times 10^{-3}$	5.71×10^{-2}
			Two-sided Kolmogorov–Smirnov test	9.71×10^{-1}	$d = 5.34 \times 10^{-3}$	-
	FSI of face units (Duyck 2021; Conv5, n = 465)	FSI of shuffled response (Duyck 2021; Conv5, n = 465)	One-sided Rank-sum test	3.01×10^{-37}	$r_{rbc} = 6.20 \times 10^{-1}$	1.00
Figure S5						
f	Effective range of population response (Translation, n = 100)	Effective range of single unit response (Translation, n = 25,043)	Two-sided Rank-sum test	1.19×10^{-24}	$r_{rbc} = 6.46 \times 10^{-2}$	1.00
	Effective range of single unit response (Translation, n = 25,043)	Effective range of image correlation (Translation, n = 1)	One-sided Signed-rank test	4.95×10^{-18}	$r_{rbc} = 1.59 \times 10^{-2}$	1.00
	Effective range of population response (Scaling, n = 100)	Effective range of single unit response (Scaling, n = 25,043)	Two-sided Rank-sum test	2.12×10^{-58}	$r_{rbc} = 1.02 \times 10^{-1}$	1.00
	Effective range of single unit response (Scaling, n = 25,043)	Effective range of image correlation (Scaling, n = 1)	One-sided Signed-rank test	1.00×10^{-20}	$r_{rbc} = 1.37 \times 10^{-1}$	1.00
	Effective range of population response (Rotation, n = 100)	Effective range of single unit response (Rotation, n = 25,043)	Two-sided Rank-sum test	1.18×10^{-34}	$r_{rbc} = 7.74 \times 10^{-2}$	1.00

	Effective range of single unit response (Rotation, n = 25,043)	Effective range of image correlation (Rotation, n = 1)	One-sided Signed-rank test	7.77×10^{-24}	$r_{rbc} = 8.77 \times 10^{-2}$	1.00
Figure S6						
a	Sigma of tuning curve in untrained network (Translation, n = 100)	Sigma of tuning curve in monkey IT (Translation, n = 1)	Two-sided Signed-rank test	6.19×10^{-1}	$r_{rbc} = -5.14 \times 10^{-2}$	6.36×10^{-2}
b	Sigma of tuning curve in untrained network (Scaling, n = 100)	Sigma of tuning curve in monkey IT (Scaling, n = 1)	Two-sided Signed-rank test	6.93×10^{-1}	$r_{rbc} = 4.12 \times 10^{-2}$	5.97×10^{-2}
Figure S7						
b	Response to face stimulus in untrained networks (n = 200)	Response to inverted face stimulus in untrained networks (n = 200)	Two-sided Rank-sum test	4.69×10^{-12}	$r_{rbc} = 3.46 \times 10^{-1}$	1.00
	Response to inverted face stimulus in untrained networks (n = 200)	Response to non-face stimulus in untrained networks (n = 200)	Two-sided Rank-sum test	5.50×10^{-28}	$r_{rbc} = 5.49 \times 10^{-1}$	1.00
c	Response to face stimulus in monkey IT (n = 64)	Response to inverted face stimulus in monkey IT (n = 64)	Two-sided Rank-sum test	2.61×10^{-22}	$r_{rbc} = 8.62 \times 10^{-1}$	1.00
	Response to inverted face stimulus in monkey IT (n = 64)	Response to non-face stimulus in monkey IT (n = 16)	Two-sided Rank-sum test	7.63×10^{-10}	$r_{rbc} = 6.93 \times 10^{-1}$	1.00
Figure S8						
a	Response of viewpoint-specific units (0˚) to each viewpoint (number of groups = 5, n = 10; number of units = 26)		One-way ANOVA	< 0.05	$f^2 \geq 3.82 \times 10^{-2}$	$\geq 4.63 \times 10^{-1}$
			Bonferroni adjustment	< 0.05	$f^2 \geq 5.42 \times 10^{-2}$	$\geq 4.81 \times 10^{-1}$
b	Response of viewpoint-invariant units to each viewpoint (number of group = 5, n = 10; number of units = 241)		One-way ANOVA	> 0.05	$f^2 \leq 3.62 \times 10^{-2}$	$\leq 4.41 \times 10^{-1}$
			Two-sided Kolmogorov–Smirnov test	$\geq 3.10 \times 10^{-2}$	$d \leq 2.69 \times 10^{-1}$	-
			Bonferroni adjustment	> 0.05	$f^2 \leq 3.13 \times 10^{-2}$	$\leq 4.34 \times 10^{-1}$
c	Weight from Conv4 face units to Conv5 0˚ specific units (n = 100)	Weight from Conv4 units to Conv5 units (n = 1)	One-sided Signed-rank test	3.02×10^{-16}	$r_{rbc} = 7.78 \times 10^{-1}$	1.00
	Weight from Conv4 all other units to Conv5 0˚ specific units (n = 100)	Weight from Conv4 units to Conv5 units (n = 1)	One-sided Signed-rank test	9.49×10^{-1}	$r_{rbc} = -7.41 \times 10^{-2}$	1.17×10^{-3}
			One-sided Kolmogorov–Smirnov test	5.30×10^{-1}	$d = 6.51 \times 10^{-1}$	-
	Weight from Conv4 face units to Conv5 0˚ specific units (n = 100)	Weight from Conv4 all other units to Conv5 0˚ specific units (n = 100)	Two-sided Rank-sum test	2.55×10^{-24}	$r_{rbc} = 7.21 \times 10^{-1}$	1.00
	Weight from Conv4 -90˚ specific units to Conv5 0˚ specific units (n = 100)	Weight from Conv4 units to Conv5 units (n = 1)	One-sided Signed-rank test	2.73×10^{-5}	$r_{rbc} = 2.96 \times 10^{-1}$	9.97×10^{-1}
	Weight from Conv4 -45˚ specific units to Conv5 0˚ specific units (n = 100)	Weight from Conv4 units to Conv5 units (n = 1)	One-sided Signed-rank test	4.59×10^{-5}	$r_{rbc} = 3.33 \times 10^{-1}$	9.93×10^{-1}
	Weight from Conv4 0˚ specific units to Conv5 0˚ specific units (n = 100)	Weight from Conv4 units to Conv5 units (n = 1)	One-sided Signed-rank test	2.62×10^{-12}	$r_{rbc} = 6.11 \times 10^{-1}$	1.00
	Weight from Conv4 45˚ specific units to Conv5 0˚ specific units (n = 100)	Weight from Conv4 units to Conv5 units (n = 1)	One-sided Signed-rank test	7.53×10^{-3}	$r_{rbc} = 2.90 \times 10^{-1}$	7.22×10^{-1}
	Weight from Conv4 90˚ specific units to Conv5 0˚ specific units (n = 100)	Weight from Conv4 units to Conv5 units (n = 1)	One-sided Signed-rank test	7.82×10^{-3}	$r_{rbc} = 1.59 \times 10^{-1}$	6.00×10^{-1}
	Weight from Conv4 0˚ specific units to Conv5 0˚ specific units (n = 100)	Weight from Conv4 specific units without 0˚ to Conv5 0˚ specific units (n = 100)	Two-sided Rank-sum test	$\leq 4.14 \times 10^{-3}$	$r_{rbc} \geq 2.03 \times 10^{-1}$	$\geq 9.55 \times 10^{-1}$
d	Weight from Conv4 face units to Conv5 invariant units (n = 100)	Weight from Conv4 units to Conv5 units (n = 1)	One-sided Signed-rank test	1.98×10^{-18}	$r_{rbc} = 9.26 \times 10^{-1}$	1.00
	Weight from Conv4 all other units to Conv5 invariant units (n = 100)	Weight from Conv4 units to Conv5 units (n = 1)	One-sided Signed-rank test	1.00	$r_{rbc} = -5.56 \times 10^{-1}$	2.17×10^{-25}

			One-sided Kolmogorov–Smirnov test	8.88×10^{-1}	$d = 2.83 \times 10^{-2}$	-
	Weight from Conv4 face units to Conv5 invariant units (n = 100)	Weight from Conv4 all other units to Conv5 invariant units (n = 100)	Two-sided Rank-sum test	3.16×10^{-34}	$r_{rbc} = 8.65 \times 10^{-1}$	1.00
	Weight from Conv4 -90° specific units to Conv5 invariant units (n = 100)	Weight from Conv4 units to Conv5 units (n = 1)	One-sided Signed-rank test	5.05×10^{-15}	$r_{rbc} = 7.41 \times 10^{-1}$	1.00
	Weight from Conv4 -45° specific units to Conv5 invariant units (n = 100)	Weight from Conv4 units to Conv5 units (n = 1)	One-sided Signed-rank test	1.08×10^{-13}	$r_{rbc} = 7.59 \times 10^{-1}$	1.00
	Weight from Conv4 0° specific units to Conv5 invariant units (n = 100)	Weight from Conv4 units to Conv5 units (n = 1)	One-sided Signed-rank test	2.31×10^{-14}	$r_{rbc} = 6.67 \times 10^{-1}$	1.00
	Weight from Conv4 45° specific units to Conv5 invariant units (n = 100)	Weight from Conv4 units to Conv5 units (n = 1)	One-sided Signed-rank test	2.12×10^{-9}	$r_{rbc} = 4.63 \times 10^{-1}$	1.00
	Weight from Conv4 90° specific units to Conv5 invariant units (n = 100)	Weight from Conv4 units to Conv5 units (n = 1)	One-sided Signed-rank test	1.88×10^{-11}	$r_{rbc} = 6.11 \times 10^{-1}$	1.00
	Weight from Conv4 specific units to each viewpoint to Conv5 invariant units (number of group = 5, n = 100)		One-way ANOVA	3.35×10^{-1}	$f^2 = 8.39 \times 10^{-5}$	8.00×10^{-2}
			Two-sided Kolmogorov–Smirnov test	$\geq 1.93 \times 10^{-1}$	$d \leq 2.13 \times 10^{-2}$	-
			Bonferroni adjustment	> 0.05	$f^2 \leq 2.67 \times 10^{-4}$	$\leq 3.08 \times 10^{-1}$
g	Viewpoint invariance index of Conv3 face units (n = 100)	Viewpoint invariance index of Conv4 face units (n = 100)	Two-sided Rank-sum test	2.89×10^{-34}	$r_{rbc} = 8.65 \times 10^{-1}$	1.00
	Viewpoint invariance index of Conv4 face units (n = 100)	Viewpoint invariance index of Conv5 face units (n = 100)	Two-sided Rank-sum test	3.78×10^{-34}	$r_{rbc} = 8.64 \times 10^{-1}$	1.00
h	Ratio of Conv3 invariant units (n = 100)	Ratio of Conv4 invariant units (n = 100)	Two-sided Rank-sum test	3.78×10^{-7}	$r_{rbc} = 3.50 \times 10^{-1}$	1.00
	Ratio of Conv4 invariant units (n = 100)	Ratio of Conv5 invariant units (n = 100)	Two-sided Rank-sum test	6.00×10^{-6}	$r_{rbc} = 3.21 \times 10^{-1}$	1.00
Figure S9						
b	Weight from Conv4 face units to Conv5 face units (n = 100)	Weight from Conv4 units to Conv5 units (n = 1)	One-sided Signed-rank test	1.97×10^{-18}	$r_{rbc} = 9.26 \times 10^{-1}$	1.00
	Weight from Conv4 other units to Conv5 face units (n = 100)	Weight from Conv4 units to Conv5 units (n = 1)	One-sided Signed-rank test	1.00	$r_{rbc} = -3.70 \times 10^{-1}$	6.54×10^{-17}
			One-sided Kolmogorov–Smirnov test	7.63×10^{-1}	$d = 4.24 \times 10^{-2}$	-
	Weight from Conv4 face units to Conv5 face units (n = 100)	Weight from Conv4 other units to Conv5 face units (n = 100)	Two-sided Rank-sum test	2.64×10^{-34}	$r_{rbc} = 8.66 \times 10^{-1}$	1.00
d	Number of Conv5 face units in untrained network (n = 100)	Expected number of Conv5 face units in untrained network (n = 100)	Two-sided Rank-sum test	3.56×10^{-1}	$r_{rbc} = 6.56 \times 10^{-2}$	5.71×10^{-1}
			Two-sided Kolmogorov–Smirnov test	1.78×10^{-3}	$d = 3.68 \times 10^{-2}$	-
h	Number of Conv5 invariant units in untrained network (n = 100)	Expected number of Conv5 invariant units in untrained network (n = 100)	Two-sided Rank-sum test	3.55×10^{-1}	$r_{rbc} = -6.56 \times 10^{-2}$	6.01×10^{-2}
			Two-sided Kolmogorov–Smirnov test	3.44×10^{-1}	$d = 1.84 \times 10^{-2}$	-
	Number of Conv5 invariant units in untrained network (n = 100)	Number of Conv5 invariant units in pretrained network (n = 1)	One-sided Signed-rank test	1.98×10^{-18}	$r_{rbc} = 9.26 \times 10^{-1}$	1.00
Figure S11						
b	Performance of Test1 (Translation, n = 100)	Performance of Test2 (Translation, n = 100)	Two-sided Rank-sum test	8.98×10^{-1}	$r_{rbc} = 9.18 \times 10^{-3}$	5.00×10^{-2}
			Two-sided Kolmogorov–Smirnov test	9.61×10^{-1}	$d = 2.02 \times 10^{-1}$	-

	Performance of Test2 (Translation, n = 100)	Performance of response shuffled (Translation, n = 100)	Two-sided Rank-sum test	2.55×10^{-34}	$r_{rbc} = 8.66 \times 10^{-1}$	1.00
	Performance of Test1 (Scaling, n = 100)	Performance of Test2 (Scaling, n = 100)	Two-sided Rank-sum test	9.45×10^{-1}	$r_{rbc} = 5.02 \times 10^{-3}$	5.00×10^{-2}
			Two-sided Kolmogorov–Smirnov test	6.77×10^{-1}	$d = 2.43 \times 10^{-1}$	-
	Performance of Test2 (Scaling, n = 100)	Performance of response shuffled (Scaling, n = 100)	Two-sided Rank-sum test	2.55×10^{-34}	$r_{rbc} = 8.66 \times 10^{-1}$	1.00
	Performance of Test1 (Rotation, n = 100)	Performance of Test2 (Rotation, n = 100)	Two-sided Rank-sum test	9.71×10^{-1}	$r_{rbc} = -2.60 \times 10^{-3}$	5.00×10^{-2}
			Two-sided Kolmogorov–Smirnov test	7.94×10^{-1}	$d = 1.82 \times 10^{-1}$	-
	Performance of Test2 (Rotation, n = 100)	Performance of response shuffled (Rotation, n = 100)	Two-sided Rank-sum test	2.55×10^{-34}	$r_{rbc} = 8.66 \times 10^{-1}$	1.00
Figure S12						
b	Performance using all selective units (n = 100)	Performance using non-face-selective units (n = 100)	Two-sided Rank-sum test	8.74×10^{-1}	$r_{rbc} = 1.14 \times 10^{-2}$	8.72×10^{-2}
			Two-sided Kolmogorov–Smirnov test	9.61×10^{-1}	$d = 1.80 \times 10^{-1}$	-
	Performance using non-face-selective units (n = 100)	Performance using face-selective units (n = 100)	Two-sided Rank-sum test	4.37×10^{-1}	$r_{rbc} = 5.52 \times 10^{-2}$	1.18×10^{-1}
			Two-sided Kolmogorov–Smirnov test	9.92×10^{-1}	$d = 1.25 \times 10^{-1}$	-
	Performance using face-selective units (n = 100)	Performance using non-selective units (n = 100)	Two-sided Rank-sum test	1.37×10^{-34}	$r_{rbc} = 8.70 \times 10^{-1}$	1.00
	Performance using non-selective units (n = 100)	Performance using shuffled response (n = 100)	Two-sided Rank-sum test	3.84×10^{-1}	$r_{rbc} = 6.18 \times 10^{-2}$	9.09×10^{-2}
			Two-sided Kolmogorov–Smirnov test	9.94×10^{-2}	$d = 1.51 \times 10^{-1}$	-

Supplementary Table 3: Summary of statistical tests on supplementary material

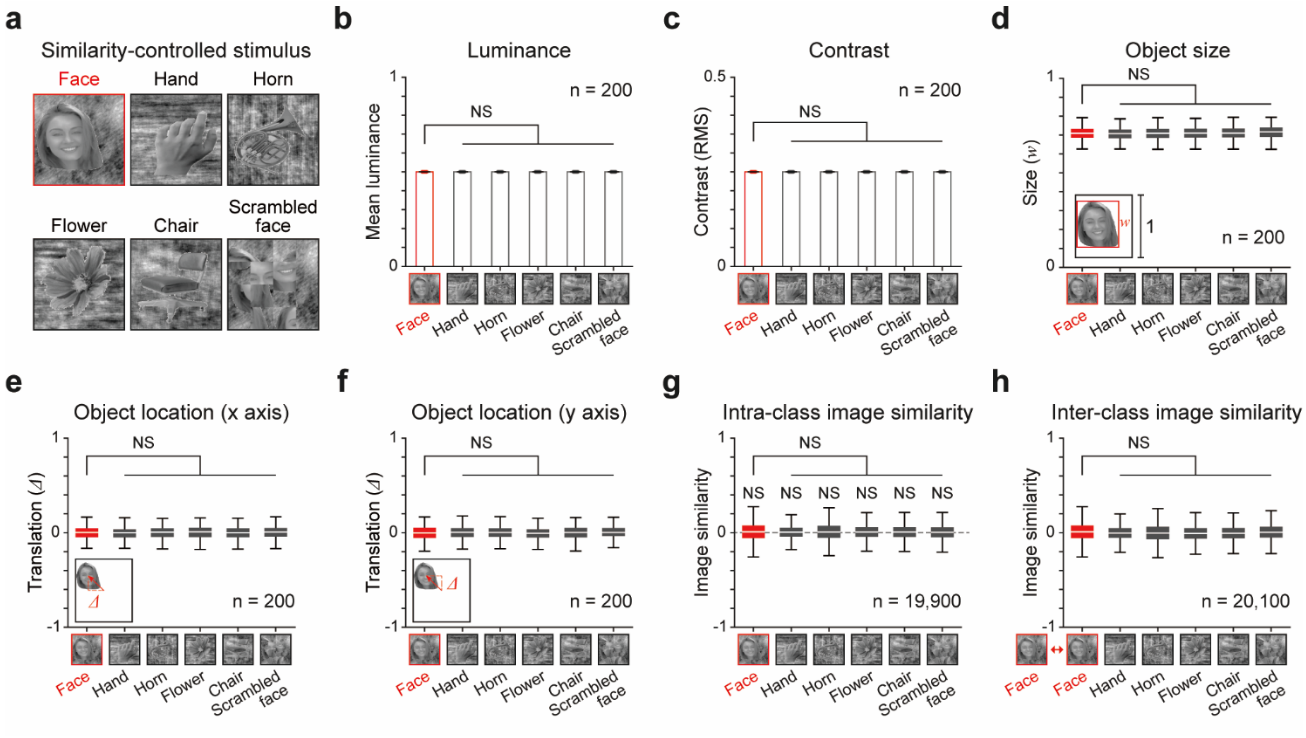

Supplementary Figure 1 | Test stimulus sets of controlled low-level visual features

A stimulus set is designed to control intra-class similarity by modifying a publicly available dataset that has been used in human fMRI study[1]. The original images are available at [http://vpnl.stanford.edu/fLoc/]. **a.** The set consists of a face, four non-face and a scrambled face class of images. **b.** Controlled luminance of the images in each class (n = 200, two-sided rank-sum test, P = 1.00). **c.** Controlled RMS contrast of images (n = 200, two-sided rank-sum test, P = 1.00). **d.** Controlled size of objects in the images in each class (n = 200, two-sided rank-sum test, $P \geq 3.22\times10^{-1}$, $r_{rbc} \leq 4.96\times10^{-2}$, two-sided Kolmogorov–Smirnov test, $P \geq 2.98\times10^{-1}$, $d \leq 4.53\times10^{-2}$). **e.** Controlled x-position of the objects in the images (n = 200, two-sided rank-sum test, $P \geq 5.16\times10^{-1}$, $r_{rbc} \leq -2.74\times10^{-3}$, two-sided Kolmogorov–Smirnov test, $P \geq 4.78\times10^{-1}$, $d \leq 3.74\times10^{-2}$). **f.** Controlled y-position of objects (n = 200, two-sided rank-sum test, $P \geq 4.29\times10^{-1}$, $r_{rbc} \leq 3.96\times10^{-2}$, two-sided Kolmogorov–Smirnov test, $P \geq 4.83\times10^{-1}$, $d \leq 4.57\times10^{-2}$). **g.** The intra-class image similarity was measured as the image correlation between images in each class (n = 19,900, two-sided rank-sum test, $P \geq 2.98\times10^{-1}$, $r_{rbc} \leq -3.81\times10^{-4}$, two-sided Kolmogorov–Smirnov test, $P \geq 3.04\times10^{-1}$, $d \leq 5.19\times10^{-2}$). **h.** The inter-class image similarity as a pixelwise image correlation between images in different class (n_{face} = 19,900, $n_{non\text{-}face}$ = 20,100, two-sided rank-sum test, $P \geq 5.50\times10^{-1}$, $r_{rbc} \leq 3.01\times10^{-3}$, two-sided Kolmogorov–Smirnov test, $P \geq 5.67\times10^{-1}$, $d \leq 3.57\times10^{-2}$). All box plots indicate the inter-quartile range (IQR between Q1 and Q3) of the dataset, the horizontal line depicts the median and the whiskers correspond to the rest of the distribution (Q1-1.5*IQR, Q3 + 1.5*IQR).

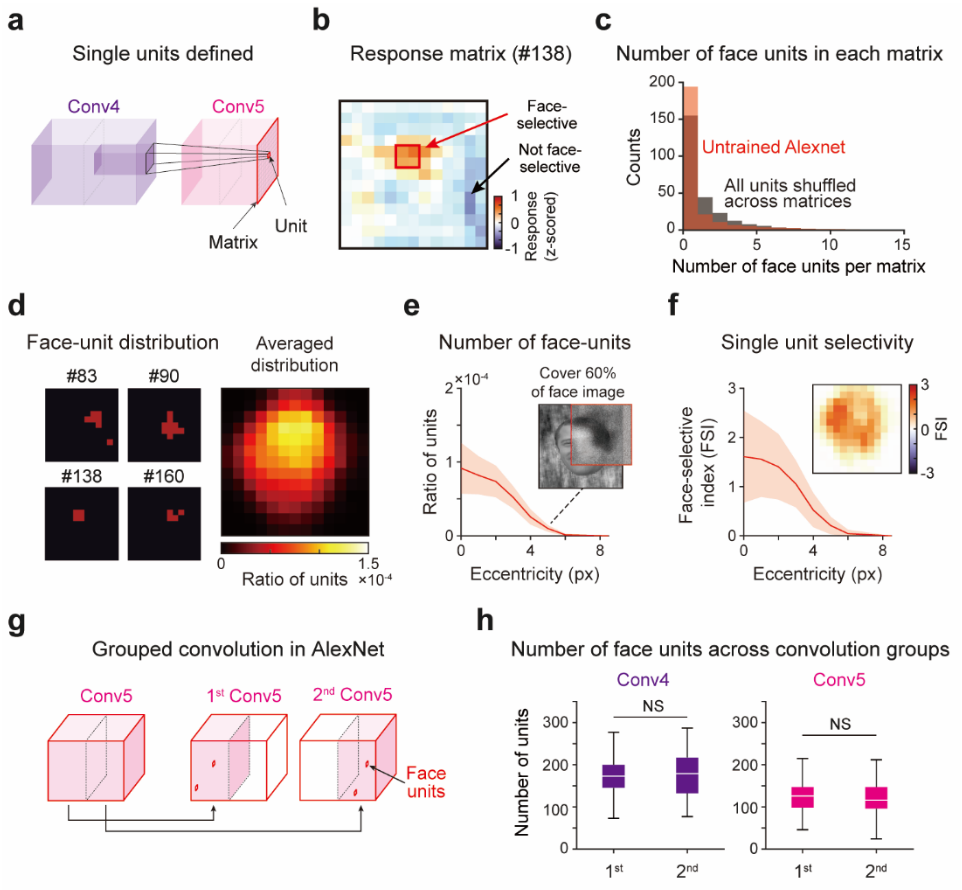

Supplementary Figure 2 | Unit distribution across filters and convolutional groups

a. Schematic of definition of unit and matrix in network. **b.** Face-selective and non-face-selective responses from the entire response matrix of Conv5 units. No filters show face-selectivity in all spatial positions. In all cases of response matrices, only a small number of face-selective units are observed for each filter. **c**. Histogram of the counted number of face-selective units observed for each filter. **d**. (Left) Distribution of face-selective units from different channels, (Right) average distribution of units across channels. **e**. The number of face-selective units with eccentricity. (Inset) The face-selective unit located at the most peripheral position has an RF that covers more than 60% of face images at the center. The shaded area indicates the standard deviation of 100 random networks. The face image in the inset is a sample stimulus from the similarity-controlled image set from the publicly available dataset[1]. The original images are available at [http://vpnl.stanford.edu/fLoc/]. **f**. Face-selectivity index with eccentricity. The shaded area indicates the standard deviation of 100 random networks. **g**. Schematics of the grouped convolution structure in AlexNet. **h**. The number of face-selective units in each convolutional group of Conv4 and Conv5 (1st Conv4 vs. 2nd Conv4, n_{Net} = 100, two-sided rank-sum test, NS, P = 4.60×10^{-1}, r_{rbc} = 5.25×10^{-2}, two-sided Kolmogorov-Smirnov test, NS, P = 2.61×10^{-1}, d = 7.83×10^{-2}; 1st Conv5 vs. 2nd Conv5, n_{Net} = 100, two-sided rank-sum test, NS, P = 5.44×10^{-1}, r_{rbc} = 4.31×10^{-2}, two-sided Kolmogorov-Smirnov test, NS, P = 5.56×10^{-1}, d = 3.02×10^{-2}). All box plots indicate the inter-quartile range (IQR between Q1 and Q3) of the dataset, the horizontal line depicts the median and the whiskers correspond to the rest of the distribution (Q1-1.5*IQR, Q3 + 1.5*IQR).

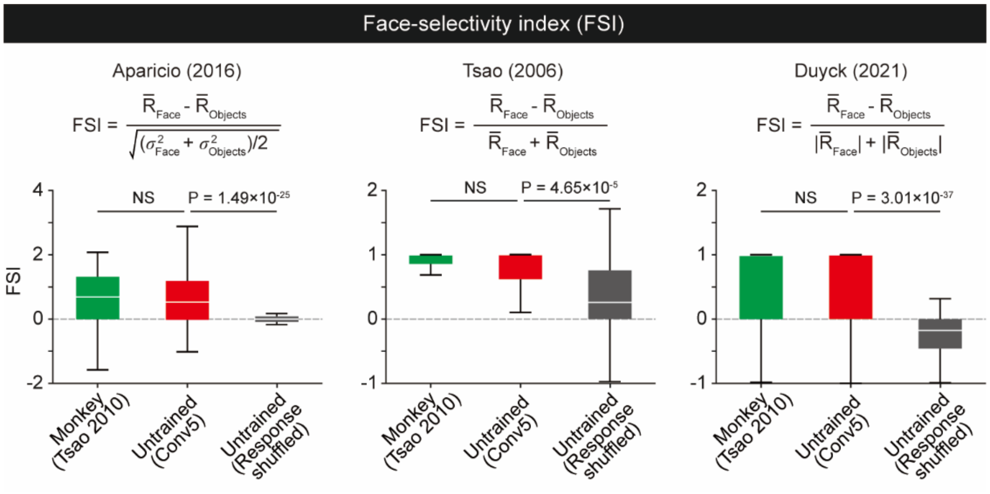

Supplementary Figure 3 | Face tuning in untrained networks for various definitions of the face selectivity index

Face-selectivity index (FSI) of face-selective neurons in the primate IT (Inferior Temporal cortex)[2] (n = 158), and the untrained AlexNet (n = 465) was estimated using definitions used in Aparicio et al., (2016)[3], Tsao et al., (2006)[4], and Duyck et al., (2021)[5]. The FSI of untrained networks was comparable to those associated with the monkey IT neurons[2] and a significantly higher value than those measured from a shuffled response (n_{monkey} = 158, $n_{untrained}$ = 465, $n_{shuffled}$ = 465, Untrained vs. Shuffled response, two-sided rank-sum test, NS, $P \geq 7.69\times10^{-2}$, $r_{rbc} \leq 9.25\times10^{-2}$, two-sided Kolmogorov-Smirnov test, NS, $P \geq 2.49 \times10^{-4}$, $d \leq 2.32\times10^{-2}$; Untrained vs. Shuffled response, two-sided rank-sum test, $P \leq 4.65\times10^{-5}$, $r_{rbc} \geq 2.10\times10^{-1}$) for various definitions of the FSI. All box plots indicate the inter-quartile range (IQR between Q1 and Q3) of the dataset, the horizontal line depicts the median and the whiskers correspond to the rest of the distribution (Q1-1.5*IQR, Q3 + 1.5*IQR).

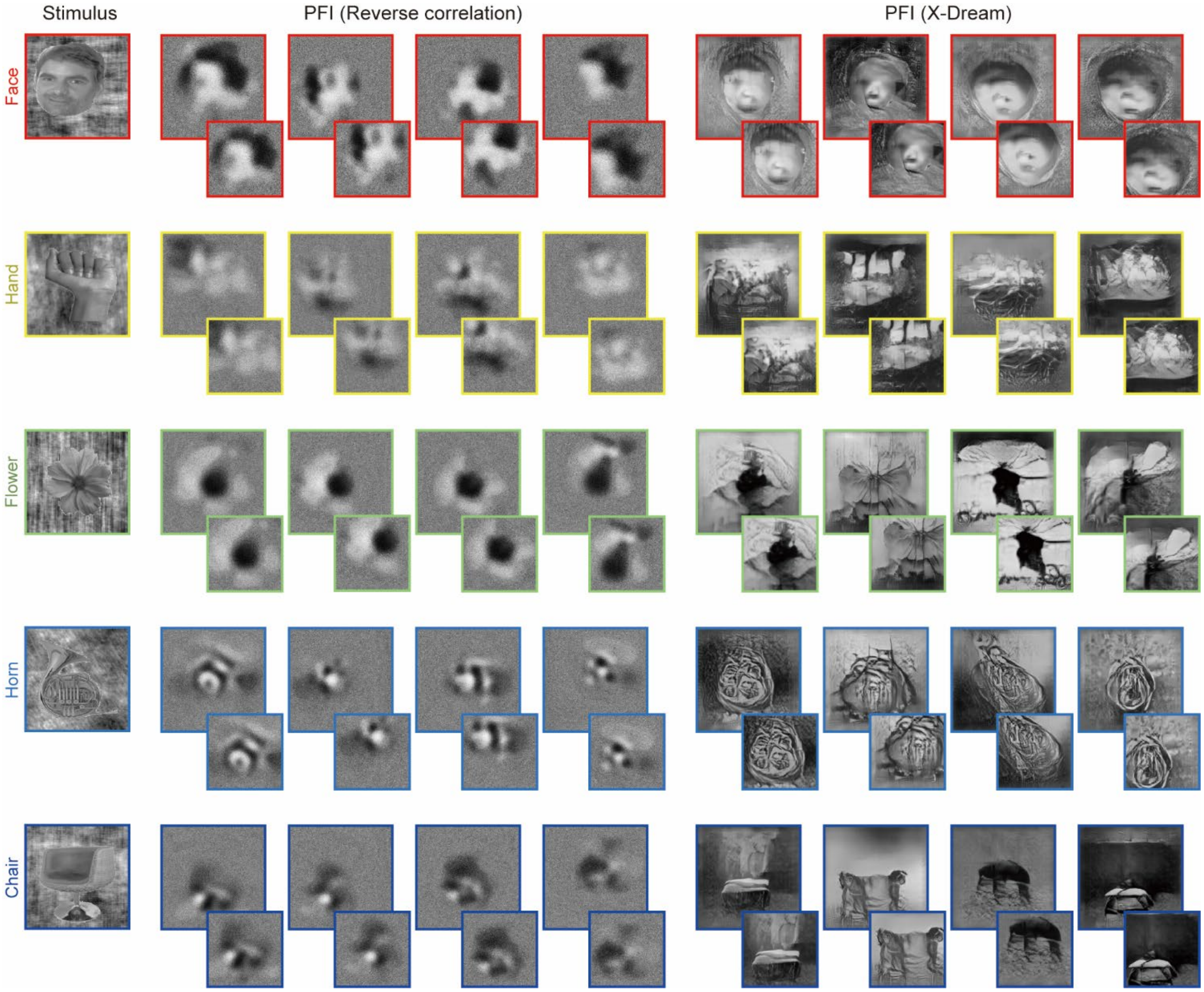

Supplementary Figure 4 | Preferred feature images of units selective for various objects

Samples of stimulus images from the similarity-controlled dataset[1]. The original images are available at [http://vpnl.stanford.edu/fLoc/]. (left) and preferred feature images (PFI) obtained using the reverse-correlation method (middle) and X-Dream (right) of face-selective units and units selective to a non-face class. (Inset) PFIs superimposed with the RFs of the corresponding units. PFIs of face-selective units represent the face-like configurations in both the reverse correlation method and X-Dream. Similarly, some of the PFIs of units selective to non-face classes show noticeable configurations of each object class.

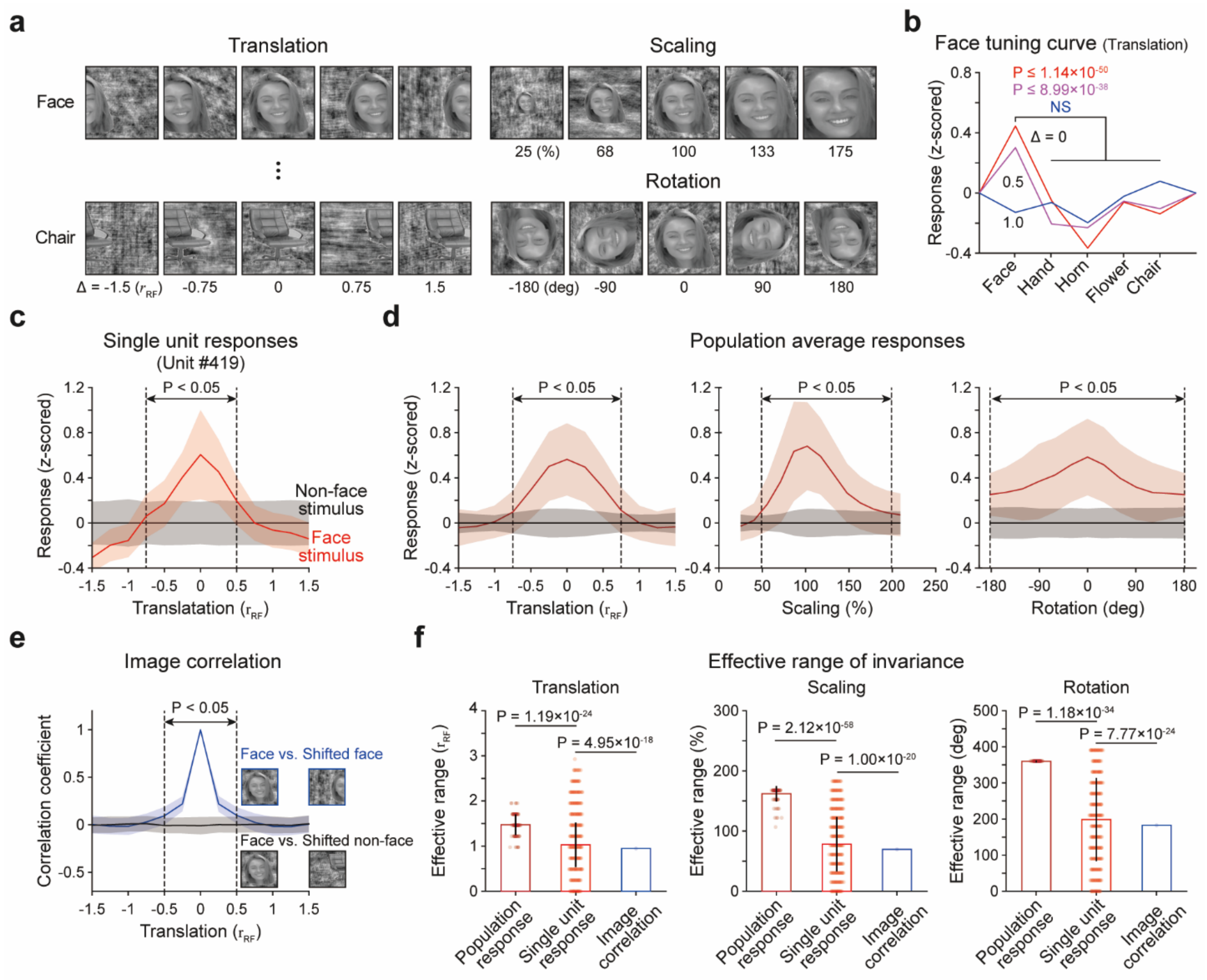

Supplementary Figure 5 | Invariant characteristics of face-selective units in untrained networks

To investigate whether the observed face-selective units show invariant representations of face images regardless of the corresponding image condition, we measured the responses of face-selective units to face and non-face object images with various positions, sizes, and rotation angles. **a.** The position, size, rotation-variant dataset was generated by translation (positions change), scaling (size change), and rotation of face and non-face objects. **b.** We found that tuning curve of a single unit shows invariance to positional translation ($n = 200$, two-sided rank-sum test, $P < 0.001$, uncorrected). **c.** We observed that single face units show constant face tuning under a fairly wide range of size/position/rotation variations ($n = 200$, one-sided rank-sum test, $P < 0.05$, uncorrected). Sample tuning profile of a single face-selective unit was shown. The red line indicates the response to face images and the black line indicates the maximum response level to non-face images. The black dashed line indicates the boundary of the position variation around which face tuning is lost. The shaded area indicates the standard deviation of 200 images. **d.** We confirmed that the invariance was also observed in the population response for which we averaged the responses of all face-selective units in an untrained network. Average tuning curves of the population responses ($n = 465$) of face-selective units to translation (Left), scaling (Middle), and rotation (Right) were shown. The shaded

area indicates the standard deviation of 200 images. To examine the effective range of the observed invariance in the face unit responses quantitatively, we estimated the boundary of the size/position/rotation variation around which face tuning is lost. We found that translation invariance was retained when the positional shift was within approximately 75% of the receptive field radius (left, one-sided rank-sum test, n = 200, $P < 0.05$, uncorrected). This indicates that these units show consistent face tuning within their receptive fields. Similarly, scaling invariance was retained when the original images (100%) were scaled from 60% to 190% (middle). Rotation invariance was retained when the original images were rotated by any angle (from -180 to 180 degrees) (right). **e.** We compared the effective range of invariance using the single unit responses of face-selective units, that of population responses, and that estimated from the pixel-wise raw image correlation between original faces and faces with low-level features varied: In this case, the effective range of the image correlation was defined as the range in which the image correlation between an original face and a transformed face image is significantly larger than that estimated between the original face and transformed non-face images (n = 200, one-sided rank-sum test, $P < 0.05$, uncorrected). The panel shows pixel-wise image correlation between the original and the transformed face images (blue) and correlation between the original and transformed non-face images as a control (black). The shaded area indicates the standard deviation of 200 images. **f.** As a result, we found that a single unit responses show a narrower effective range of invariance to object translation/scaling/rotation compared to that of population average responses (Population vs. Single, $n_{pop} = 100$, $n_{single} = 25{,}043$, two-sided rank-sum test, $P \leq 1.19 \times 10^{-24}$, $r_{rbc} \geq 6.46 \times 10^{-2}$), whereas both the population responses and the single unit responses still showed a significantly wider effective range of translation/scaling/rotation invariance compared to that of the raw image pixel correlation (Single vs. Image correlation, $n_{single} = 25{,}043$, $n_{corr} = 1$, one-sided signed-rank test, $P \leq 4.95 \times 10^{-18}$, $r_{rbc} \geq 1.59 \times 10^{-2}$). The effective ranges of invariant responses to size, position, and rotational variations were shown in panel. The error bar indicates the standard deviation of 100 random networks. Stimulus images in panel (a) and (e) are generated from the similarity-controlled image dataset[1]. The original images are available at [http://vpnl.stanford.edu/fLoc/].

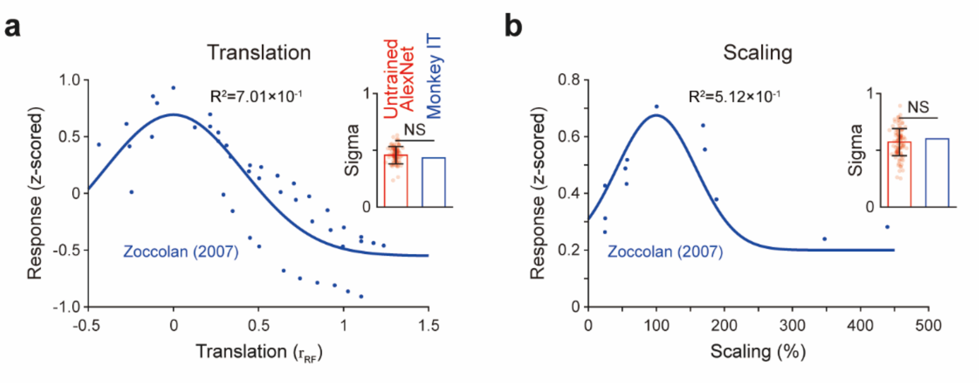

Supplementary Figure 6 | Invariant face-selective units in untrained networks and monkey IT

a. We found that the effective range of the invariant response shows values comparable to those measured in neurons in the monkey IT (Inferior Temporal cortex)[6]. The panel shows translation-invariant responses of neurons observed in the monkey IT[6]. The measured responses were fitted to a Gaussian distribution (R^2 = 7.01×10^{-1}, blue solid line). (Inset) The sigma of a Gaussian function fitted to neural responses in the monkey IT (blue) and that in untrained networks (red) ($n_{untrained}$ = 100, n_{monkey} = 1, two-sided signed-rank test, NS, P = 6.19 ×10^{-1}, r_{rbc} = -5.14 ×10^{-2}). **b.** Scale-invariant neural responses in the monkey IT[6], fitted to a Gaussian distribution (R^2 = 5.12×10^{-1}, blue solid line). (Inset) The sigma of a Gaussian function fitted to neural responses in the monkey IT (blue) and in untrained networks (red) ($n_{untrained}$ = 100, n_{monkey} = 1, two-sided signed-rank test, NS, *P* = 6.93 ×10^{-1}, r_{rbc} = 4.12 ×10^{-2}). All error bars indicate the standard deviation of 100 random networks.

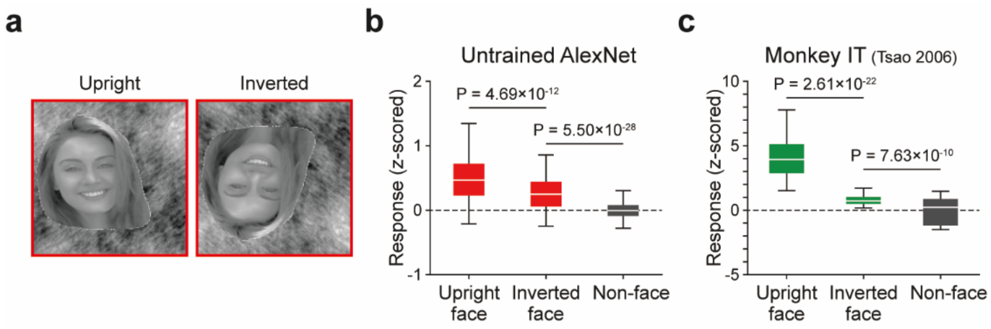

Supplementary Figure 7 | Inversion effect in untrained networks

We found that our model units also show the inversion effect observed in monkeys[4,7,8]. We found that the responses of face-selective units to inverted face images are significantly lower than those to upright faces, whereas these responses are still higher than those to non-face images. **a.** The panel shows sample images of upright and inverted faces, which are generated from the similarity-controlled dataset[1]. The original images are available at [http://vpnl.stanford.edu/fLoc/]. **b.** The panel shows the inversion effect observed in untrained networks. The response of face-selective units to upright face and inverted face was measured in untrained network (Upright vs. Inverted, n = 200, two-sided rank-sum test, $P = 4.69 \times 10^{-12}$, $r_{rbc} = 3.46 \times 10^{-1}$; Inverted vs. Non-face, n = 200, two-sided rank-sum test, $P = 5.50 \times 10^{-28}$, $r_{rbc} = 5.49 \times 10^{-1}$). **c**. The panel shows the inversion effect observed in the monkey IT (Inferior Temporal cortex)[4] (Upright vs. Inverted, n = 64, two-sided rank-sum test, $P = 2.61 \times 10^{-22}$, $r_{rbc} = 8.62 \times 10^{-1}$; Inverted vs. Non-face, $n_{inverted} = 64$, $n_{non\text{-}face} = 16$, two-sided rank-sum test, $P = 7.63 \times 10^{-10}$, $r_{rbc} = 6.93 \times 10^{-1}$). These results indicate that our face-selective units show similar responses to upright and inverted faces, i.e. the "inversion effect", as observed in the monkey IT[4]. All box plots indicate the inter-quartile range (IQR between Q1 and Q3) of the dataset, the horizontal line depicts the median and the whiskers correspond to the rest of the distribution (Q1-1.5*IQR, Q3 + 1.5*IQR).

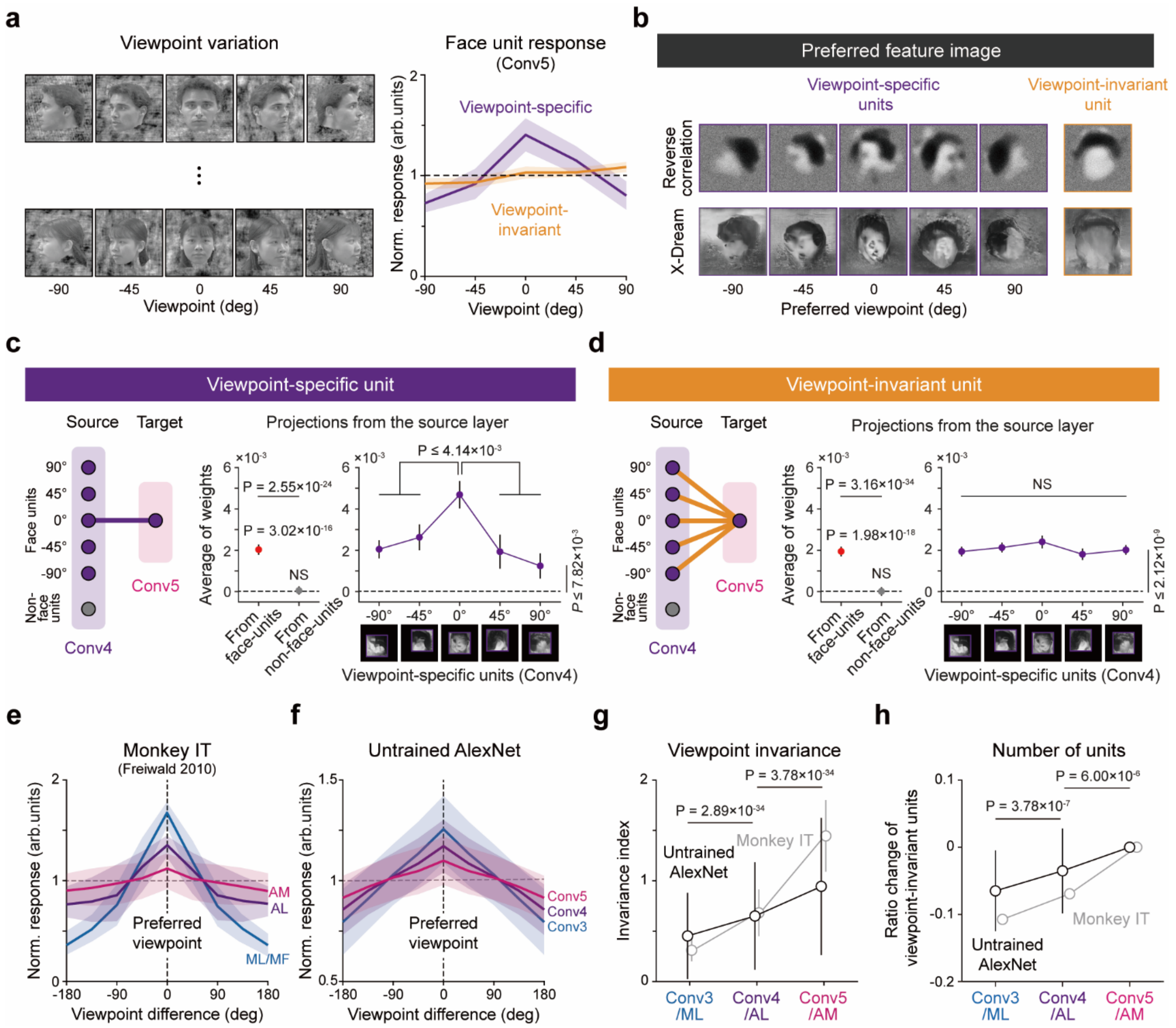

Supplementary Figure 8 | Increasing viewpoint invariance of face-selective units along the network hierarchies

In previous studies involving monkeys, it was observed that face-selective neurons in the inferior temporal cortex (IT) show responses invariant to diverse angles of face images, a condition called viewpoint invariance[2]. It was reported that there are subpopulations of face-selective neurons which show invariant characteristics to various viewpoints of face images, i.e., viewpoint-invariant neurons, and sub-populations of face-selective neurons which show selective responses to a specific viewpoint of face images, i.e., viewpoint-specific neurons. **a.** To investigate whether these viewpoint-invariant characteristics can also be observed in an untrained network, we measured the responses of Conv5 in an untrained AlexNet while face images at different viewpoints were provided to the network. We found that there is a viewpoint-invariant population whose responses are not significantly different across all viewpoint classes (one-way ANOVA, $P > 0.05$, Bonferroni adjustment n=5) and a viewpoint-specific population

whose responses are significantly different (one-way ANOVA, $P < 0.05$, Bonferroni adjustment n=5), as observed in the monkey IT[2]. The panel shows face images with various viewpoints that are selected from the publicly available Point' 04 dataset[9]. The original images are available at [http://crowley-coutaz.fr/Head%20Pose%20Image%20Database.html]. (Left), viewpoint-invariant and the viewpoint-specific responses of face-selective units (Right). The shaded area indicates the standard deviation of 10 images. **b.** The average PFI of these viewpoint-specific units selective for each viewpoint reveals the representative feature of a face rotated at a specific angle: an asymmetric hairline with only one visible eye for 45-degree specific face units and a symmetric hairline with clearly shown eyes and nose for center-specific face units. The panel shows the PFIs of viewpoint-specific and viewpoint-invariant units in Conv5 in the untrained networks. Based on this result, we hypothesized that viewpoint-invariant units may arise from the projection of multiple viewpoint-specific units preferring various viewpoints in the previous layer. **c.** To validate this scenario, we backtracked projections of the units from the source layer (Conv4) to the target layer (Conv5) and examined the weights of connected viewpoint-specific units. First, we confirmed that viewpoint-specific ($n = 136 \pm 30$) and viewpoint-invariant face-selective units ($n = 163 \pm 35$) exist in Conv4, as well as in Conv5 (viewpoint-specific, $n = 96 \pm 27$, viewpoint-invariant, $n = 127 \pm 36$). We found that the viewpoint-specific units in Conv5 receive inputs from Conv4 units strongly biased to a particular viewpoint angle (right, two-sided rank-sum test, $P \leq 4.14\times10^{-3}$, $r_{rbc} \geq 2.03\times10^{-3}$). The error bar indicates the standard error of 100 random networks. The panel presents the weight values of the projections from Conv4 units to a viewpoint-specific unit in the Conv5 target layer. **d.** However, the viewpoint-invariant units in Conv5 receive input from Conv4 units with a fairly homogeneous distribution of viewpoint angles (right, one-way ANOVA with Bonferroni adjustment n=5, NS, $P = 3.35\times10^{-1}$, $f^2 = 8.39\times10^{-5}$, two-sided Kolmogorov–Smirnov test, $P \geq 1.93\times10^{-1}$, $d \leq 2.12\times10^{-2}$). The error bar indicates the standard error of 100 random networks. The panel presents the weight values of the projections from Conv4 units to a viewpoint-invariant unit in Conv5. The PFI of viewpoint-specific units in Conv4 connected to viewpoint-invariant units in Conv5 (as shown (b)) also show whole face-configuration at each viewpoint angle. These results suggest that the viewpoint-invariant units in Conv5 as well as viewpoint-specific units connected to them are selective to whole faces rather than face parts. **e**. In a previous study focusing on the monkey IT, it was observed that higher up in the hierarchy in the IT (from the middle lateral (ML) to the anterior medial (AM) area), neurons show an increasing trend toward invariance. The panel shows average tuning curves of face-selective neurons in each layer, which reveals increasing viewpoint-invariant characteristics in the monkey IT[2]. The response was normalized to the average value as 1. Shaded area indicates the standard error of neurons in each IT hierarchy. **f**. In a subsequent analysis, we found that such layer-specific characteristics of viewpoint invariance also emerge in an untrained network, reproducing viewpoint-invariant profiles, consistent with that observed in biological data[2]. We found that the level of invariance increased along the network hierarchy in an untrained AlexNet as observed in the monkey IT[2]. The panel shows average tuning curves of face-selective units in each layer, which reveals increasing viewpoint-invariant characteristics along the untrained AlexNet. Shaded area indicates the standard error of 100 random networks. **g.** To quantify these invariant characteristics, we introduced an invariance index of units, defined as the inverse of the standard deviation of responses across different viewpoints. The panel shows the increase of the invariance index of face-selective neurons higher up the hierarchy in the monkey IT[2] and in the untrained AlexNet.

As a result, we found that higher layers (Conv4 and Conv5) show greater invariance compared to that in lower layers (Conv3) (Conv3 vs. Conv4, n_{Net} = 100, two-sided rank-sum test, $P = 2.89 \times 10^{-34}$, $r_{rbc} = 8.65 \times 10^{-1}$, Conv4 vs. Conv5, n_{Net} = 100, two-sided rank-sum test, $P = 3.78 \times 10^{-34}$, $r_{rbc} = 8.64 \times 10^{-1}$). Each dot indicates the mean and error bar indicates the standard error of 100 simulations of randomly initialized networks. **h.** Increase in the number of invariant units higher up in the hierarchy. We found that the number of invariant units is also increased along the hierarchy, as observed in the monkey IT (Conv3 vs. Conv4, n_{Net} = 100, two-sided rank-sum test, $P = 3.78 \times 10^{-7}$, $r_{rbc} = 3.50 \times 10^{-1}$, Conv4 vs. Conv5, n_{Net} = 100, two-sided rank-sum test, $P = 6.00 \times 10^{-6}$, $r_{rbc} = 3.21 \times 10^{-1}$). Each dot indicates the mean and error bar indicates the standard deviation of 100 untrained networks.

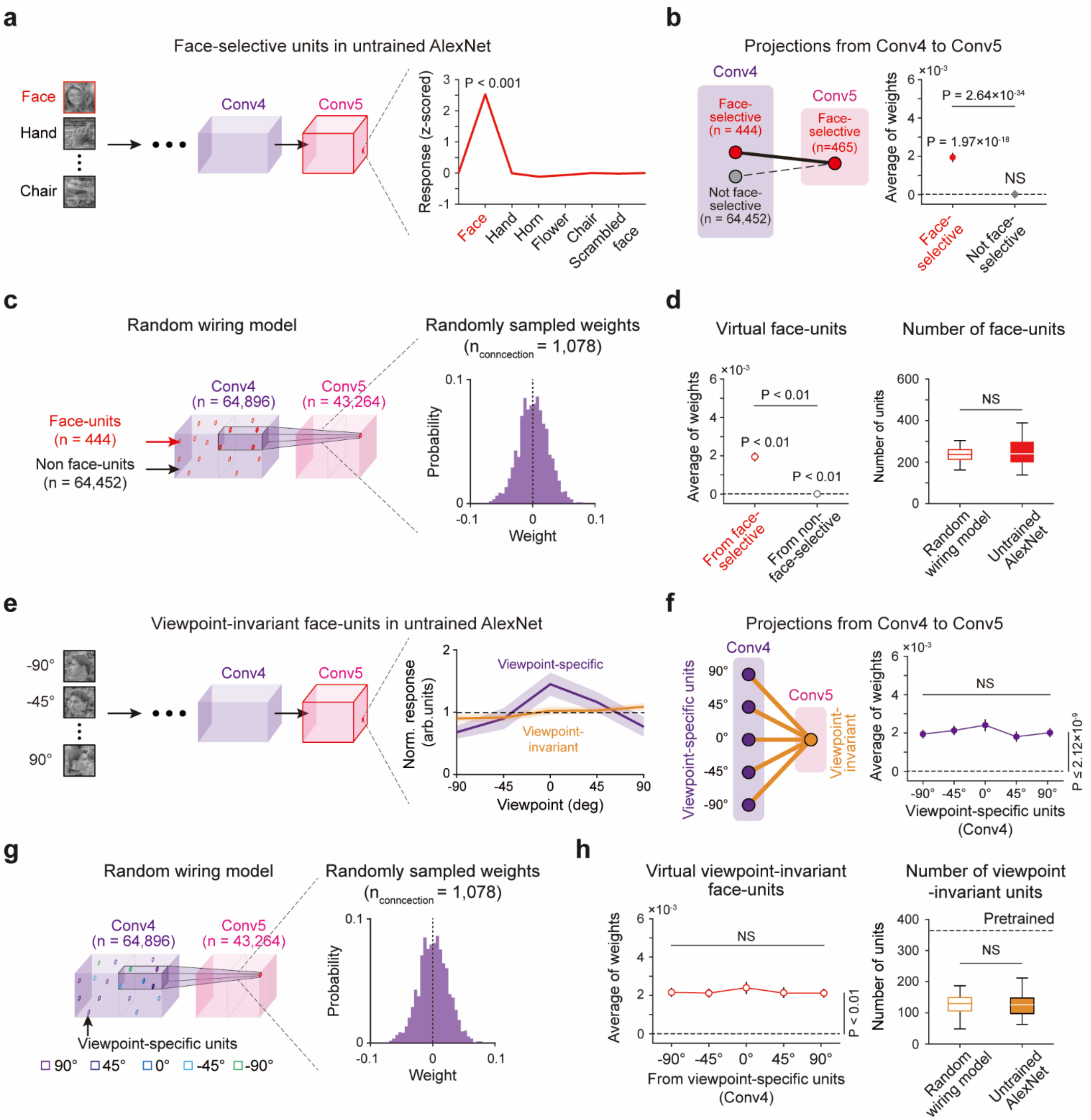

Supplementary Figure 9 | Viewpoint invariance emerged by statistical variance in random network

a. Face-selective units observed in Conv4 and Conv5 of untrained networks (P < 0.001, two-sided rank-sum test, uncorrected). Stimulus images shown in this panel are sampled from the similarity-controlled dataset[1]. The original images are available at [http://vpnl.stanford.edu/fLoc/]. **b**. Face-selective units in Conv5 of untrained networks are observed to receive strong projections from face-selective units in Conv4 (right, n_{Net} = 100). **c**. A random wiring model in which face-selective units in Conv5 emerge from the random wiring of face-selective units in Conv4: The numbers of face units and non-face units are counted in Conv4, and the numbers of viewpoint-specific units were

counted separately. To model the random wiring from Conv4 to Conv5, random weight values (n=1,078; matching the size of the convolutional filter) were sampled and applied between randomly sampled Conv4 units and a model unit in Conv5. This process was repeated to generate N=43,264 model neurons in Conv5. **d**. The connectivity from units in Conv4 to each model unit in Conv5 was examined. Conv5 units that receive stronger connections from face units in Conv4 compared to those from non-face units (one-sided rank-sum test, $P < 0.01$) were labeled as virtual face-selective units in Conv5 (left). The number of these virtual face-selective units was counted in Conv5. The number of emerging virtual face-selective units by random wiring is consistent with face-selective units observed in untrained networks (right, $n_{Net} = 100$, two-sided rank-sum test, NS, $P = 3.56\times10^{-1}$, $r_{rbc} = 6.56\times10^{-2}$, two-sided Kolmogorov–Smirnov test, $P = 1.78\times10^{-3}$, $d = 3.68\times10^{-2}$). **e**. Viewpoint-specific and viewpoint-invariant face units in Conv4 and Conv5 of untrained networks were selected according to their responses to a stimulus at each viewpoint: The responses of viewpoint-invariant units are not significantly different across viewpoint angles (one-way ANOVA, $P > 0.05$, Bonferroni adjustment n=5), while those of viewpoint-specific units to the preferred viewpoint are significantly higher than those at any other angle ($P < 0.05$). The shaded area indicates the standard deviation of 10 images. **f**. The weight projections from Conv4 viewpoint-specific units to a Conv5 viewpoint-invariant unit show a uniform distribution across preferred viewpoints (one-way ANOVA, $P > 0.05$, Bonferroni adjustment n=5). The error bar indicates the standard error of 100 random networks. Stimulus images shown in this panel are sampled from the viewpoint dataset, modified from the publicly available Point' 04 dataset[9]. The original images are available at [http://crowley-coutaz.fr/Head%20Pose%20Image%20Database.html]. **g**. To test whether the observed uniform weight distribution can develop by chance in random networks, a random wiring model in which face-selective units in Conv5 that emerge from the random wiring of face-selective units in Conv4 was simulated. **h**. (Left) A Conv5 unit is virtually considered as viewpoint-invariant if the projected feedforward weights from Conv4 viewpoint-specific units are uniform (one-way ANOVA, $P > 0.05$, Bonferroni adjustment n=5). The error bar indicates the standard error of 100 random networks. (Right). The number of units with a uniform weight distribution (virtual viewpoint-invariant units) that emerged from random wiring in the model matches that observed in untrained networks ($n_{Net} = 100$, two-sided rank-sum test, NS, $P = 3.55\times10^{-1}$, $r_{rbc} = -6.56\times10^{-2}$, two-sided Kolmogorov–Smirnov test, $P = 3.44\times10^{-1}$, $d = 1.84\times10^{-2}$). Notably, the number of viewpoint-invariant units is greater in pre-trained networks than in untrained networks (one-sided signed-rank test, $P = 1.98\times10^{-18}$, $r_{rbc} = 9.26\times10^{-1}$). All box plots indicate the inter-quartile range (IQR between Q1 and Q3) of the dataset, the horizontal line depicts the median and the whiskers correspond to the rest of the distribution (Q1-1.5*IQR, Q3 + 1.5*IQR).

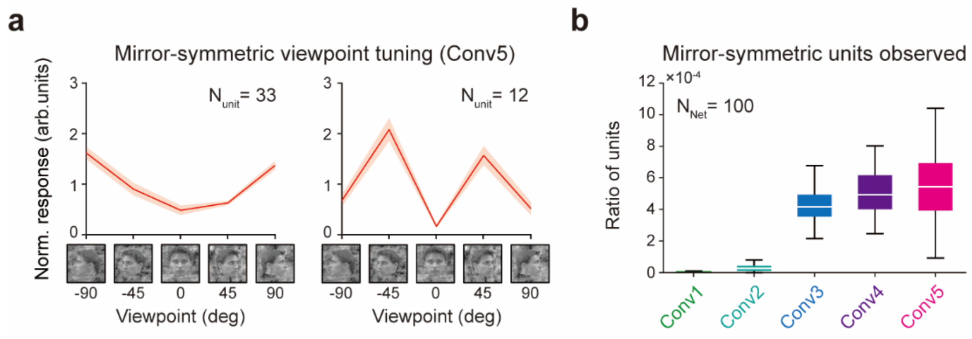

Supplementary Figure 10 | Mirror-symmetric tuning of face units

a. We investigated whether mirror-symmetric tuning, another interesting characteristic of face neurons observed in the monkey IT (Inferior Temporal cortex)[2], can arise in our untrained networks. To do this, we defined mirror-symmetric tuning as a condition in which a viewpoint-specific face-selective unit has a symmetric shape of the tuning curve (i.e., a unit shows peak responses at -45˚ and 45˚ or -90˚ and 90˚). From the repeated simulations of 100 randomly initialized untrained networks, we observed 24 ± 9 mirror-symmetric units out of 43,264 units in Conv5. The panel shows examples of mirror-symmetric tuning of face units. The response was normalized to the average value as 1. The shaded area indicates the standard error of mirror-symmetric units. Stimulus images shown in this panel are sampled from the viewpoint dataset, modified from the publicly available Point' 04 dataset[9]. The original images are available at [http://crowley-coutaz.fr/Head%20Pose%20Image%20Database.html]. **b.** The number of mirror-symmetric units (n_{Net} = 100, Conv1: n = 0.0002 ± 0.0003%, Conv2: n = 0.003 ± 0.002%, Conv3: n = 0.043 ± 0.011%, Conv4: n = 0.051 ± 0.014%, Conv5: n = 0.055 ± 0.021%). All box plots indicate the inter-quartile range (IQR between Q1 and Q3) of the dataset, the horizontal line depicts the median and the whiskers correspond to the rest of the distribution (Q1-1.5*IQR, Q3 + 1.5*IQR).

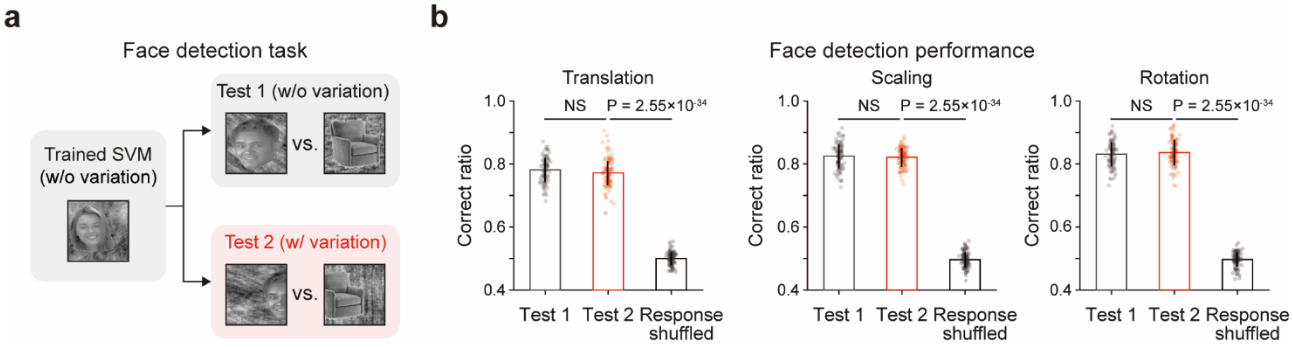

Supplementary Figure 11 | Face detection performance with variation of the low-level features

We hypothesized that the invariant properties of face units may enable the networks to detect faces with various sizes, positions, and rotations even when such low-level features were held constant when training the SVM classifier. **a.** To test this idea, we trained the SVM with the network responses of face-selective units without variation of the low-level features described above. The panel shows an illustration of the training and test sessions for the feature-variant face detection task. In the training session, the SVM was trained with network responses for faces with no feature variation. In the test session, the trained SVM was tested with a (1) test set including faces without feature variations, and (2) a test set including faces with feature variations. Images for training and test 1 shown in this panel are sampled from the similarity-controlled dataset, modified from the publicly available dataset[1]. Images for test 2 in this panel are sampled from the feature-variant dataset, modified from the publicly available dataset[1]. The original images are available at [http://vpnl.stanford.edu/fLoc/]. **b.** We found that the detection performance of test set 2 with position variation is comparable to that of the test set 1 (left**,** Test1 vs. Test2, n_{Net} = 100, NS, two-sided rank-sum test, $P = 8.98\times10^{-1}$, $r_{rbc} = 9.18\times10^{-3}$, two-sided Kolmogorov-Smirnov test, NS, $P = 9.61\times10^{-1}$, $d = 2.02\times10^{-1}$; Test2 vs. Response shuffled, two-sided rank-sum test, $P = 2.55\times10^{-34}$, $r_{rbc} = 8.66\times10^{-1}$). Similarly, we obtained the same results with test set 2 with variations of the size (middle, Test1 vs. Test2, n_{Net} = 100, two-sided rank-sum test, NS, $P = 9.45\times10^{-1}$, $r_{rbc} = 5.02\times10^{-3}$, two-sided Kolmogorov-Smirnov test, NS, $P = 6.77\times10^{-1}$, $d = 2.43\times10^{-1}$; Test2 vs. Response shuffled, two-sided rank-sum test, $P = 2.55\times10^{-34}$, $r_{rbc} = 8.66\times10^{-1}$) and rotation (right, Test1 vs. Test2, n_{Net} = 100, two-sided rank-sum test, NS, $P = 9.71\times10^{-1}$, $r_{rbc} = -2.60\times10^{-3}$, two-sided Kolmogorov-Smirnov test, NS, $P = 7.94\times10^{-1}$, $d = 1.82\times10^{-1}$; Test2 vs. Response shuffled, two-sided rank-sum test, $P = 2.55\times10^{-34}$, $d = 8.66\times10^{-1}$). These results indicate the face-selective units can perform invariant detection of faces regardless of feature variations of the faces. Each bar indicates the mean and error bar indicates the standard deviation of 100 simulations of randomly initialized networks.

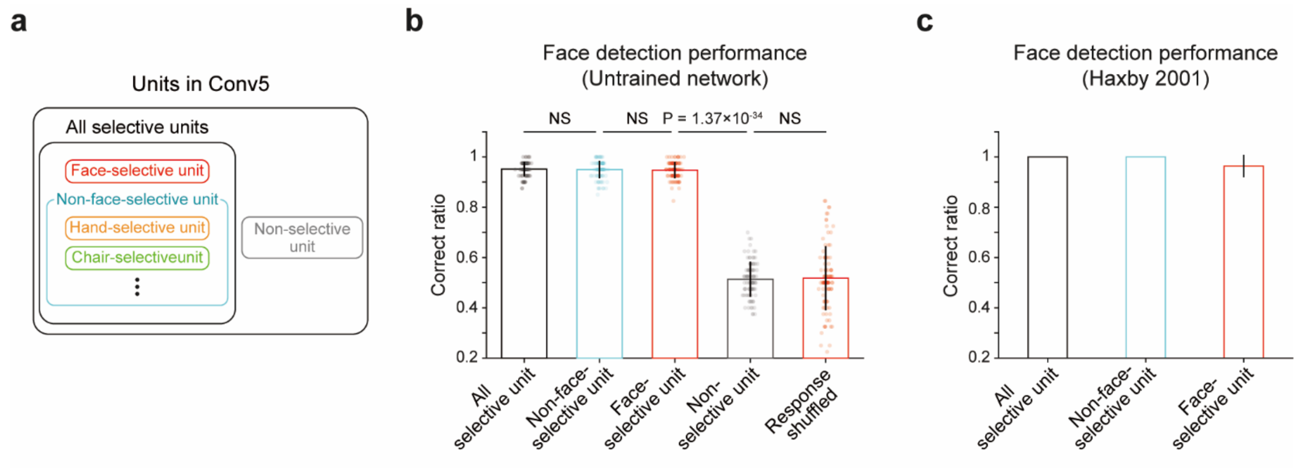

Supplementary Figure 12 | Face detection performance of selective units in untrained networks

a. Distinct types of selective units in Conv5: (1) all selective units, (2) units selective to non-face classes (nonface-selective), (3) face-selective units, (4) units selective to none of these classes (non-selective). **b.** Simulated face detection performance using distinct types of selective units in Conv5 and using the shuffled responses of face-selective units in untrained networks (All selective vs. non-face-selective, n_{trial} = 100, two-sided rank-sum test, NS, P = 8.74×10^{-1}, r_{rbc} = 1.14×10^{-2}, two-sided Kolmogorov-Smirnov test, NS, P = 9.61×10^{-1}, d = 1.80×10^{-1}; non-face-selective vs. face-selective, n_{trial} = 100, two-sided rank-sum test, NS, P = 4.37×10^{-1}, r_{rbc} = 5.52×10^{-2}, two-sided Kolmogorov-Smirnov test, NS, P = 9.92×10^{-1}, d = 1.25×10^{-1}; face-selective vs non-selective, n_{trial} = 100, two-sided rank-sum test, P = 1.37×10^{-34}, r_{rbc} = 8.70×10^{-1}; non-selective vs shuffled, n_{trial} = 100, two-sided rank-sum test, NS, P = 3.84×10^{-1}, r_{rbc} = 6.18×10^{-2}, two-sided Kolmogorov-Smirnov test, NS, P = 9.94×10^{-2}, d = 1.51×10^{-1}). Each bar indicates the mean and error bar indicates the standard deviation of 100 repeated trials. **c.** Face detection performance previously reported in human study[10] using (1) all selective neurons, (2) nonface-selective neurons, and (3) face-selective neurons.

Supplementary References

1. Stigliani, A., Weiner, K. S. & Grill-Spector, K. Temporal processing capacity in high-level visual cortex is domain specific. *J. Neurosci.* **35**, 12412–12424 (2015).
2. Freiwald, W. A. & Tsao, D. Y. Functional compartmentalization and viewpoint generalization within the macaque face-processing system. *Science.* **330**, 845–851 (2010).
3. Aparicio, P. L., Issa, E. B. & DiCarlo, J. J. Neurophysiological organization of the middle face patch in macaque inferior temporal cortex. *J. Neurosci.* **36**, 12729–12745 (2016).
4. Tsao, D. Y., Freiwald, W. A., Tootell, R. B. H. & Livingstone, M. S. A cortical region consisting entirely of face-selective cells. *Science.* **311**, 670–674 (2006).
5. Duyck, M. *et al.* Color tuning of face-selective neurons in macaque inferior temporal cortex. *eNeuro* **8**, 1–16 (2021).
6. Zoccolan, D., Kouh, M., Poggio, T. & DiCarlo, J. J. Trade-off between object selectivity and tolerance in monkey inferotemporal cortex. *J. Neurosci.* **27**, 12292–12307 (2007).
7. Buiatti, M. *et al.* Cortical route for facelike pattern processing in human newborns. *Proc. Natl. Acad. Sci. U. S. A.* **116**, 4625–4630 (2019).
8. Perrett, D. I. *et al.* Visual cells in the temporal cortex sensitive to face view and gaze direction. *Proc. R. Soc. London - Biol. Sci.* **223**, 293–317 (1985).
9. Gourier, N., Hall, D. & Crowley, J. L. Estimating face orientation from robust detection of salient facial structures. *FG Net Work. Vis. Obs. Deictic Gestures* 17–25 (2004).
10. Haxby, J. V *et al.* Distributed and overlapping representations of faces and objects in ventral temporal cortex. *Science.* **293**, 2425–2430 (2001).
